# Calmodulin Binding Activates *Chromobacterium* CopC Effector to ADP-Riboxanate Host Apoptotic Caspases

**DOI:** 10.1128/mbio.00690-22

**Published:** 2022-04-21

**Authors:** Yaxin Liu, Huan Zeng, Yanjie Hou, Zilin Li, Lin Li, Xiaocui Song, Jingjin Ding, Feng Shao, Yue Xu

**Affiliations:** a Graduate Program in Chinese Academy of Medical Sciences and Peking Union Medical College, Beijing, China; b National Laboratory of Biomacromolecules, CAS Center for Excellence in Biomacromolecules, Institute of Biophysics, Chinese Academy of Sciences, Beijing, China; c National Institute of Biological Sciences, Beijing, Beijing, China; d Research Unit of Pyroptosis and Immunity, Chinese Academy of Medical Sciences and National Institute of Biological Sciences, Beijing China; e Tsinghua Institute of Multidisciplinary Biomedical Research, Tsinghua University, Beijing, China; f Key Laboratory of Cell Differentiation and Apoptosis of Chinese Ministry of Education, Department of Pathophysiology, Shanghai Jiao Tong University School of Medicine, Shanghai, China; New York University School of Medicine

**Keywords:** ADP-riboxanation, type III secretion system (T3SS), *Chromobacterium violaceum*, apoptosis, pyroptosis, caspase, bacterial effector, posttranslational modification (PTM), CopC, OspC3

## Abstract

Blocking host cell death is an important virulence strategy employed by many bacterial pathogens. We recently reported that Shigella flexneri inhibits host pyroptosis by delivering a type III secretion system (T3SS) effector OspC3 that catalyzes a novel arginine ADP-riboxanation modification on caspase-4/11. Here, we investigated the OspC3 homologue CopC from Chromobacterium violaceum, an opportunistic but sometimes deadly bacterial pathogen. CopC bears the same arginine ADP-riboxanase activity as OspC3, but with a different substrate specificity. Through proteomic analysis, we first identified host calmodulin (CaM) as a binding partner of CopC. The analyses additionally revealed that CopC preferably modifies apoptotic caspases including caspase-7, -8 and -9. This results in suppression of both extrinsic and intrinsic apoptosis programs in C. violaceum-infected cells. Biochemical reconstitution showed that CopC requires binding to CaM, specifically in the calcium-free state, to achieve efficient ADP-riboxanation of the caspases. We determined crystal structure of the CaM-CopC-CASP7 ternary complex, which illustrates the caspase recognition mechanism and a unique CaM-binding mode in CopC. Structure-directed mutagenesis validated the functional significance of CaM binding for stimulating CopC modification of its caspase substrates. CopC adopts an ADP-ribosyltransferase-like fold with a unique His-Phe-Glu catalytic triad, featuring two acidic residues critical for site-specific arginine ADP-riboxanation. Our study expands and deepens our understanding of the OspC family of ADP-riboxanase effectors.

## INTRODUCTION

During infection, programmed cell death (PCD), such as apoptosis and pyroptosis, generally serves as a host defense to fight the intruder (1). Suicidal infected cells can obliterate pathogens in a variety of ways, including but not limited to disrupting replicative niches of the pathogen and eliciting protective immune responses. Apoptosis is the prototype of PCD and occurs via a cascade of proteolytic events mediated by the caspase family of cysteine proteases. Initiator caspase-8 and caspase-9 are provoked by extrinsic (death receptor-mediated) and intrinsic (mitochondrial) signals, respectively, and then cleave caspase-3 and/or caspase-7. Matured caspase-3/7 sunder diverse substrates and eventually lead to cell demise (2, 3). It is generally accepted that the antibacterial function of apoptosis is attributed to efferocytosis of infected cells, in which the contents of dying or dead cells are packaged into intact particles and ultimately eliminated by phagocytes. Besides direct microbial degradation, phagocytes can also present foreign antigens to T cells and prime adaptive immunity to further boost the defense (4). Pyroptosis is a type of necrotic cell death that can also limit bacterial infections (5). Various pathogen-associated molecular patterns (PAMPs) stimulate caspase-1 or caspase-4/11 activation through the inflammasome pathways. Activated caspase-1/4/11 subsequently cleave the auto-inhibited gasdermin D (GSDMD) protein and liberate its N-terminal pore-forming domain to perforate the plasma membrane (6–8). Pore formation and the resulting membrane rupture releases proinflammatory cytokines and danger signals from pyroptotic cells, which, along with lysis of intracellular bacteria, elicits potent inflammatory responses, for instance by stimulating humoral immunity (9).

Not surprisingly, successful bacterial pathogens have evolved toxins or translocated effectors to counteract PCD in the host (4, 10–12). Various pathogen-encoded cell death inhibitors have been identified and shown to play important roles in counteracting host defenses, and these inhibitors often employ a unique mechanism of action such as imposing a posttranslational modification (PTM) not present in the host. For example, enteropathogenic and enterohemorrhagic Escherichia coli (EPEC and EHEC) secrete the T3SS effector NleB that enzymatically adds an *N*-acetylglucosamine moiety to an arginine in the death domains of host death receptors and their downstream adaptors to block extrinsic apoptosis (13, 14); EPEC/EHEC also translocates NleF effector into host cells that directly binds to and inhibits apoptotic caspases (15). Chlamydia trachomatis degrades host proapoptotic BH3-only proteins by secreting a serine protease CPAF (16). YopM from *Yersinia* spp. hijacks host kinases (PKNs and RSKs) to cause hyper-phosphorylation of host Pyrin protein and impairs Pyrin inflammasome-stimulated pyroptosis (17). Recently, we reported that Shigella flexneri T3SS effector OspC3 possesses an unprecedented ADP-riboxanase activity that transfers an ADP-ribose moiety from NAD (NAD^+^) to an arginine and a subsequent deamination of the ADP-ribosylated arginine to form an oxazolidine ring (9). OspC3-catalyzed PTM, named as ADP-riboxanation, specifically targets caspase-4/-11 to block pyroptosis triggered by cytosolic lipopolysaccharides (LPS) from invading bacteria.

OspC3 homologs are present in a diverse spectrum of bacteria, many of which, including the T3SS effector CopC from Chromobacterium violaceum, display the ADP-riboxanase activity *in vitro* (9). C. violaceum is a Gram-negative, facultative anaerobic, and opportunistic pathogen, and its infection in humans causes severe abscesses in various organs with a high mortality rate (18, 19). In the murine infection model, the virulence of C. violaceum primarily relies on its pathogenicity islands 1 (Cpi-1)-encoded T3SS (20). Sixteen Cpi-1 effectors, including CopC, are identified, but their functions are largely unknown (21). Our previous study indicates that *copC*-deficient C. violaceum has decreased virulence in mice (9), suggesting a functional importance of this ADP-riboxanase effector for C. violaceum infection.

Here, we investigated the mechanism of the CopC effector. We first identified calmodulin (CaM) as a host-binding partner and cofactor of CopC. We further found that CopC is different from OspC3 and much prefers to target caspase-8, -9 and -7, rather than caspase-4/11. CopC efficiently modifies these caspases also by arginine ADP-riboxanation, causing complete inactivation of host apoptotic pathways during C. violaceum infection. The modification activity of CopC heavily relies on its binding to CaM, specifically in the calcium-free state. Finally, we solved the crystal structure of CaM-CopC-CASP7 ternary complex, and the structure reveals detailed mechanisms underlying CaM-stimulated ADP-riboxanation of the caspase by CopC. Altogether, our study establishes a unique virulence mechanism employed by a bacterial effector to target apoptotic caspases and block host cell apoptosis.

## RESULTS

### Caspase-4 is not the physiological substrate of CopC.

We recently reported that all OspC3 family members share the same domain organization, which contains an N-terminal catalytic domain and a C-terminal ankyrin repeat domain (ARD). Overexpression of various OspC3 homologues, including CopC from C. violaceum, in mammalian cells can induce the same arginine ADP-riboxanation of caspase-4/11 as that by OspC3. CopC bears about 36% identity and 56% similarity to OspC3 in primary sequences, and the residues including E326 and H328, essential for OspC3 to modify caspase-4/11, are conserved in CopC (Fig. S1 in the supplemental material). As expected, co-expression of caspase-4 (C258A, protease-deficient) with wild-type (WT) CopC but not the E325A/H327A (EH) mutant (equivalent to E326A/H328A in OspC3) led to a modification on caspase-4 that could be detected by an anti-ADP-ribose (anti-ADPr) antibody (Fig. S2A). The modification was reconstituted *in vitro* and mass spectrometry of the modified caspase-4 revealed a 524-Da mass increase on Arg314 (Fig. S2B), confirming that CopC harbors the same ADP-riboxanase activity as OspC3. We noticed that the modification by CopC on caspase-4 was much weaker than that by OspC3. When titrating amounts of purified CopC or OspC3 protein were electroporated into HeLa cells, the dose of CopC required for blocking LPS-stimulated pyroptosis was 10 times the dose of OspC3 (Fig. S2C). In the infection context, only C. violaceum that expressed exogenous CopC could induce weak modification of caspase-11 but not caspase-4 (Fig. S2D). In contrast, C. violaceum expressing OspC3 led to robust ADP-riboxanation of both caspase-4 and caspase-11 (Fig. S2D). Moreover, C. violaceum Δ*copC* infection triggered evident cleavage of GSDMD in HeLa cells, comparable to that in WT C. violaceum-infected cells, and additional expression of CopC in C. violaceum Δ*copC* did not affect the level of GSDMD cleavage (Fig. S2E). In contrast, robust cleavage of GSDMD was only observed in S. flexneri Δ*ospC3*-infected but not WT S. flexneri*-*infected cells (Fig. S2E). Thus, CopC could not efficiently modify caspase-4 and interfere with the caspase-4-GSDMD pyroptosis pathway during C. violaceum infection. We also confirmed that CopC was not involved in C. violaceum invasion into host cells (Fig. S2F). Given that *copC*-deficient C. violaceum is less virulent in mice than WT bacteria, the above analyses indicate that CopC likely targets other host substrates for ADP-riboxanation to promote bacterial virulence.

10.1128/mbio.00690-22.1 FIG S1 Sequence alignment of C. violaceum effector CopC, S. flexneri effectors OspC1, OspC2, and OspC3. ClustalW2 was used to derive the alignment displayed using ESPript 3.0. Identical residues are highlighted by dark red background and conserved residues are in red. Secondary structures of CopC are labeled and shown on top of the alignment. The putative NAD^+^-binding H-F-E triad and two catalytical residues (D172/D230) are highlighted by blue and cyan backgrounds, respectively. Download FIG S1, PDF file, 1.6 MB. Copyright © 2022 Liu et al. 2022 Liu et al. https://creativecommons.org/licenses/by/4.0/ This content is distributed under the terms of the Creative Commons Attribution 4.0 International license.

10.1128/mbio.00690-22.2 FIG S2 (A) Caspase-4 ADP-riboxanation by overexpressed CopC. 293T cells were cotransfected with 3xFlag-caspase-4 (p30 form, C258A) and 6xMyc-CopC (WT or E325A/H327A) mutant. Cell lysates were subjected to anti-Flag immunoprecipitation (Flag IP) followed by immunoblotting as indicated. Immunoblotting of total lysates (Input) shows the expression of CopC. (B) Mass spectrometry of CopC-modified caspase-4. Caspase-4 was reacted with excessive CopC *in vitro* and then subjected to c mass spectrometry. Shown is the tandem mass spectrum of the modified caspase-4 peptide _314_RDSTMGSIF. (C) HeLa cells were electroporated with LPS together with indicated amounts of purified OspC3, CopC, or MBP proteins. ATP-based cell viability was determined 2 h postelectroporation. Data are presented as means ± SD of three individual replicates. (D) 293T cells transfected with 3xFlag-caspase-4 or -11 (p30 form, C/A) were infected with C. violaceum Δ*copC* complemented with CopC, OspC3, or an empty vector. Lysates of infected cells were subjected to anti-Flag immunoprecipitation followed by immunoblotting. (E) HeLa cells stably expressing caspase-4 and GSDMD were infected with C. violaceum (WT or a *copC* deletion/complementation strain) or S. flexneri (WT or *Δosp*C3). Cell lysates were immunoblotted with anti-GSDMD antibody. GSDMD-N fragment represents the cleaved active form. (F) HeLa cells were infected with C. violaceum WT, Δ*copC*, or CopC complementary strain. Bacterial invasion was determined by counting the numbers of colonies recovered from HeLa cells at 1 h postinfection (means ± SD from three determinations). (G to I) CaM expression in C. violaceum has no effect on bacterial NAD^+^ content and growth. WT C. violaceum was transfected with an empty vector or a plasmid expressing CaM. Bacterial lysates were subjected to anti-CaM immunoblotting and Coomassie Blue-staining (loading control) (G). Overnight bacteria cultures were transferred to fresh medium (1:33), followed by optical density measurements at 600 nm (OD_600_) at the indicated time points (H). NAD^+^ contents in bacterial lysates were assayed using HPLC-MS, and relative NAD^+^ concentrations were shown as means ± SD from three determinations (I). Download FIG S2, PDF file, 0.7 MB. Copyright © 2022 Liu et al. 2022 Liu et al. https://creativecommons.org/licenses/by/4.0/ This content is distributed under the terms of the Creative Commons Attribution 4.0 International license.

### CopC binds to calmodulin and the binding stimulates CopC enzymatic activity.

**FIG 1:**
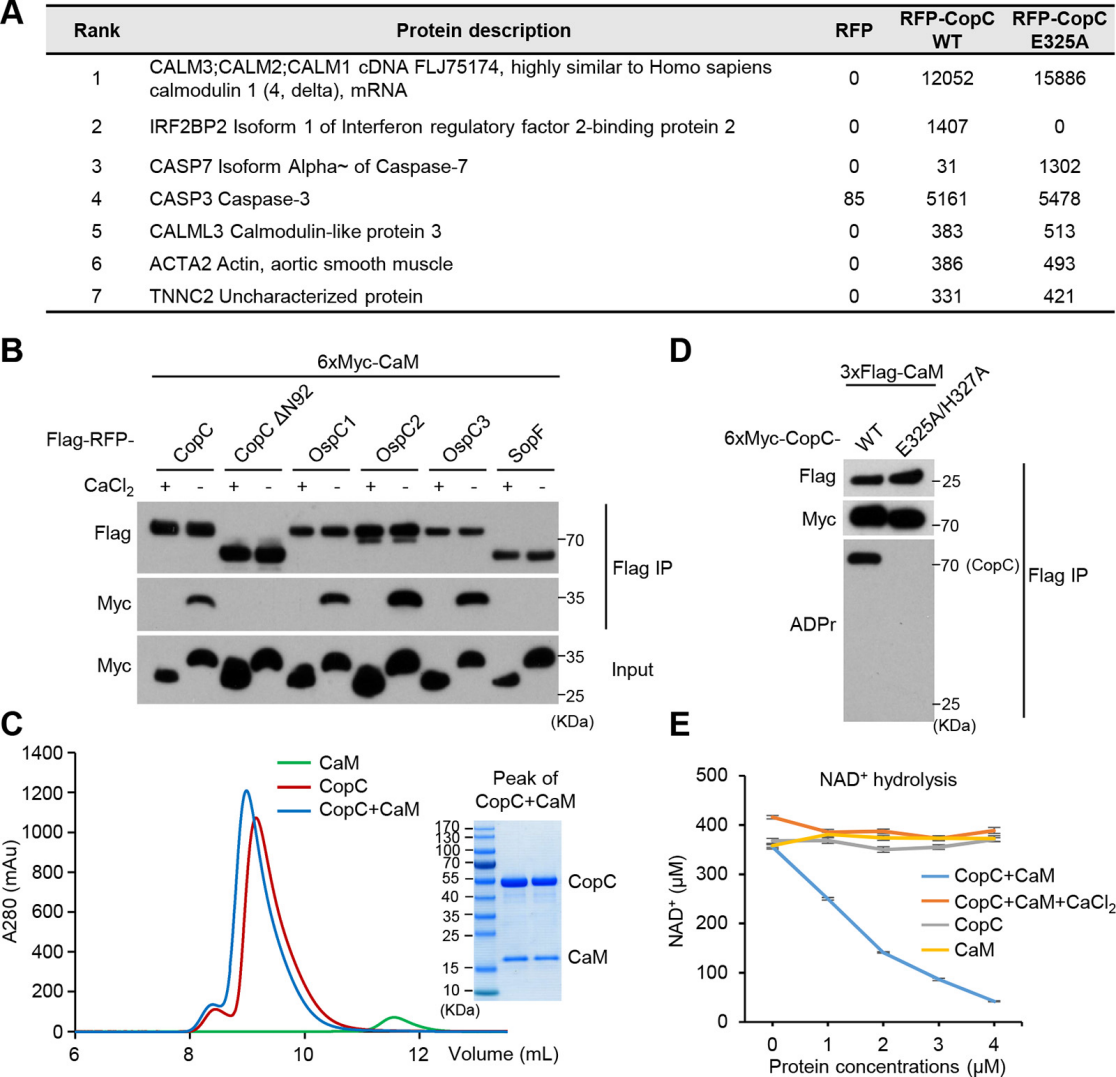
Identification of calmodulin as a host binding partner of CopC. (A) Proteomic identification of CopC-interacting proteins. HeLa cells were transiently transfected with Flag-RFP or Flag-RFP-CopC (WT or E325A). Cell lysates were subjected to anti-Flag immunoprecipitation and analyzed by mass spectrometry. The numbers are Mascot scores of each hit protein. (B) Coimmunoprecipitation interaction between OspC-family proteins and CaM. 293T cells were cotransfected with 6xMyc-CaM and an indicated Flag-RFP-tagged OspC-family protein including CopC and CopC ΔN92. Anti-Flag immunoprecipitation was performed in the presence of 5 mM CaCl_2_ or 2 mM EDTA (calcium-free). The immunoprecipitates (Flag IP) and total cell lysates (Input) were immunoblotted with indicated antibodies. Ca^2+^-bound CaM and apo CaM showed different mobility on the SDS-PAGE gel. (C) Gel-filtration chromatography analyses of CopC and CaM complex formation. To analyze complex formation, recombinant CopC and CaM proteins were mixed at the molar ratio of 1:1. Coomassie-stained SDS-PAGE gel of the peak fraction of CopC+CaM samples is shown. (D) CaM is not a substrate of CopC. 293T cells were transfected with 3xFlag-CaM and 6xMyc-CopC (WT or E325A/H327A). Anti-Flag immunoprecipitates (Flag IP) were blotted with indicated antibodies including the anti-ADPr antibody that only recognized WT CopC but not CaM. (E) NAD^+^-hydrolysis activity of the CopC-CaM complex. Consumption of 400 μM NAD^+^ (16°C, 5 h) were measured in the presence of indicated amounts of CopC or CaM protein alone, or the CopC-CaM complex (with or without 5 mM CaCl_2_).

To identify potential substrate(s) or binding partners of CopC, we transiently expressed Flag-RFP tagged CopC (WT or the catalytically inactive E325A mutant) in HeLa cells and performed mass spectrometry analyses of anti-Flag immunoprecipitates. Calmodulin (CaM) was the most enriched protein in the CopC (both WT and E325A) immunoprecipitates, but did not appear in the RFP control group (Fig. 1A). CaM regulates a wide variety of cellular processes through calcium binding-induced conformational changes (22). Co-immunoprecipitation in transfected 293T cells confirmed the specific interaction between CopC and CaM, which was inhibited by 5 mM CaCl_2_ (Fig. 1B). Of note, when the first two α-helix, according to the predicted secondary structure of CopC, were deleted, the resulting CopC ΔN92 became incapable of interacting with CaM (Fig. 1B). Similar calcium-sensitive interaction with CaM was observed with OspC3 as well as OspC1/2, but not Salmonella ADP-ribosyltransferase SopF (23) (Fig. 1B). Furthermore, recombinant CopC and CaM proteins purified from E. coli formed a stable complex in the absence of calcium with a 1:1 stoichiometry (Fig. 1C).

Despite a robust co-immunoprecipitation interaction between CaM and CopC in the 293T-cell overexpression system, no ADP-riboxanation was detected on CaM while auto-ADP-riboxanation was found on CopC (Fig. 1D). Thus, CaM is not the ADP-riboxanation substrate of CopC. Bacterial effectors often rely on binding to certain host factors for enzymatic activation or functioning. In line with this notion, incubation of recombinant CopC with CaM was found to greatly stimulate its NAD^+^ hydrolysis activity, which was blocked by 5 mM CaCl_2_ (Fig. 1E). Thus, binding to calcium-free CaM stimulates the enzymatic activity of CopC. We also measured NAD^+^ contents and growth curve of C. violaceum that expressed or did not express CaM, and found that neither bacterial NAD^+^ contents nor their growth rate were affected by enforced expression of CaM (Fig. S2G to S2I). This probably owes to the low expression of CaM or limited substrate-free NAD^+^ hydrolysis activity in CopC.

### CopC targets caspase-7, -8, and -9 for ADP-riboxanation to block apoptosis.

**FIG 2:**
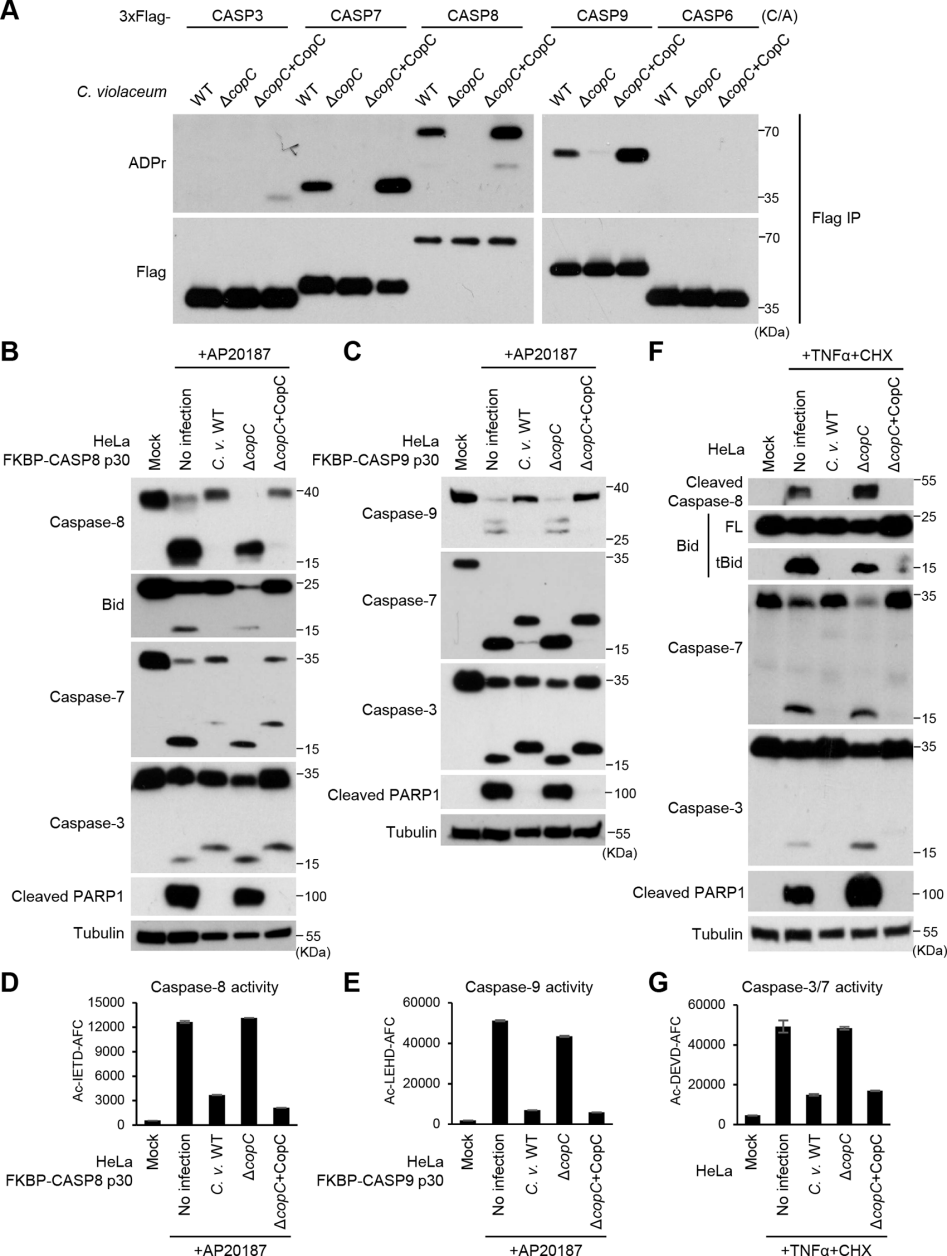
C. violaceum blocks the apoptosis pathway in a CopC-dependent manner. (A) Modification of various apoptotic caspases by CopC during C. violaceum infection. 293T cells expressing 3xFlag-caspase-3, -7, -8, -9, or -6 (the C/A mutant) were infected with C. violaceum WT, Δ*copC*, or Δ*copC* complemented with CopC. The caspases were immunoprecipitated from lysates of infected cells and blotted with anti-Flag and anti-ADP ribose antibodies as shown. (B to G) Blocking different apoptotic cascades by CopC during C. violaceum infection. HeLa cells stably expressing FKBP-CASP8 p30 (B), FKBP-CASP9 p30 (C), or intact HeLa cells (F) were infected with C. violaceum WT, Δ*copC*, or Δ*copC* complemented with CopC; 1 h postinfection, cells were treated with 100 nM AP20187 (B and C) or 30 ng/mL TNF-α plus 100 μg/mL CHX. Cell lysates were blotted with indicated antibodies against core proteins in the apoptosis pathway including caspase-3, caspase-7, Bid, and PARP1. Quantitative measurements of indicated caspase activity using their specific fluorogenic tetrapeptide substrates are shown in D, E, and G. Data are shown as means ± SD from three determinations. Ac-IETD-AFC, Ac-LEHD-AFC, and Ac-DEVD-AFC are specific substrates for caspase-8, caspase-9 and caspase-3/7, respectively.

Our proteomic analyses also identified caspase-3 and caspase-7 as the top hits of CopC-interacting proteins (Fig. 1A). Given that pyroptotic caspases are not the physiological substrates of CopC, we hypothesized that CopC might prefer to target other caspases (like caspase-3/7) for ADP-riboxanation. To test this idea, the catalytic cysteine mutants (C/A) of a series of caspases including caspasae-3, -6, -7, -8, and -9 were individually expressed in HeLa cells and the cells were then subjected to infection by WT C. violaceum, C. violaceum Δ*copC* or Δ*copC* complemented with a CopC expression plasmid. Notably, anti-ADPr immunoblotting analyses revealed that WT C. violaceum infection caused evident ADP-riboxanation of caspase-7, -8, and -9 but not caspase-3 and -6 (Fig. 2A). Modification of caspase-7/8/9 was absent in C. violaceum Δ*copC* infection, which was restored by re-expression of CopC in the Δ*copC* strain. In HeLa cells infected with the CopC-complemented (overexpression) strain, caspase-6 was still not modified, but a weak ADPr modification signal was detected on caspase-3 (Fig. 1A and 2A). Apparently, the modification capacity of CopC toward caspase-3 was modest despite a robust binding.

Caspase-8 and -9 are constrained as latent zymogens and undergo proximity-induced activation by the upstream death-inducing signaling complex (DISC) and the apoptosome complex, respectively. To investigate the effect of caspase-8/9 modification by CopC, we fused the FK506-binding protein (FKBP) with the protease domain (p30) of caspase-8 or -9 and expressed the chimeric proteins in HeLa cells. Addition of the homodimerizer AP20187 to the cells induced rapid dimerization and activation of the engineered caspases to cleave their endogenous substrates, bypassing the upstream stimuli (24, 25). Notably, C. violaceum infection efficiently blocked AP20187-induced activation of caspase-8/9, as well as their cleavage of downstream substrates Bid, caspase-7, caspase-3, and PARP1 (Fig. 2B and C). This cascade of inhibitory effects was abolished in C. violaceum Δ*copC*-infected cells, which could be rescued by re-expression of CopC in the mutant bacteria (Fig. 2B and C). CopC-mediated inactivation of caspase-8/9 during C. violaceum infection was further confirmed by directly measuring the activity of these caspases using their specific tetrapeptide substrates (Fig. 2D and E).

To further establish the function of CopC, HeLa cells were stimulated with physiological death-receptor ligands including TNF-α, TRAIL, or Fas ligand (FasL) to induce activation of endogenous caspase-8. As shown in Fig. 2F, TNF-α plus cycloheximide (CHX) treatment triggered autocleavage of caspase-8 as well as its cleavages of Bid, caspase-3, caspase-7, and PARP1, all of which was blocked by infection with CopC-positive C. violaceum but not Δ*copC* strain. Results of a similar nature were obtained in HeLa cells stimulated with two other death-receptor ligands, TRAIL and FasL (Fig. S3A and S3B). CopC-mediated inactivation of the death receptor signaling pathways was corroborated by measuring the proteolytic activity of caspase-3/7 in TNF-α plus CHX-treated and C. violaceum-infected cells (Fig. 2G). Morphological analyses confirmed that C. violaceum-secreted CopC could block TNF-α or TRAIL plus CHX-stimulated cell shrinkage and nuclear fragmentation, two phenotypic features of apoptotic cell death (Fig. S3C). Moreover, we also assayed the effect of CopC on endogenous caspase-9 activation using doxycycline (Dox)-induced expression of truncated Bid (tBid). The results showed that tBid-induced caspase-9 activation, as well as its cleavages of caspase-3, caspase-7, and PARP1, was blocked by CopC-positive C. violaceum but not the Δ*copC* infection (Fig. S3D). Thus, C. violaceum T3SS effector CopC directly modifies and inactivates caspase-8 and -9, thereby blocking both extrinsic and intrinsic apoptosis pathways.

10.1128/mbio.00690-22.3 FIG S3 (A to C) Blocking the extrinsic apoptotic pathways by CopC during C. violaceum infection. HeLa cells were infected with C. violaceum WT, Δ*copC*, or Δ*copC* complemented with CopC; 1 h postinfection, cells were stimulated with 200 ng/mL TRAIL plus 100 μg/mL CHX (A and C), 1 μg/mL FasL (B), or 30 ng/mL TNFα plus 100 μg/mL CHX (C). The immunoblotting in A and B was performed similarly as that in Fig. 2B. To examine apoptotic morphology, nuclei of fixed cells were stained with 4′,6-diamidino-2-phenylindole (DAPI). Representative fluorescent and bright field (DIC) cell images are shown (scale bar, 10 μm). (D) Blocking the intrinsic apoptotic pathway by CopC during C. violaceum infection. HeLa cells stably expressing truncated Bid (tBid) under a doxycycline (Dox)-inducible promoter were infected as in A. One hour post-infection, cells were treated with 1 μg/mL Dox and analyzed by immunoblotting as shown. Download FIG S3, PDF file, 0.9 MB. Copyright © 2022 Liu et al. 2022 Liu et al. https://creativecommons.org/licenses/by/4.0/ This content is distributed under the terms of the Creative Commons Attribution 4.0 International license.

### CaM binding promotes CopC to catalyze ADP-riboxanation of caspase-7/8/9.

**FIG 3:**
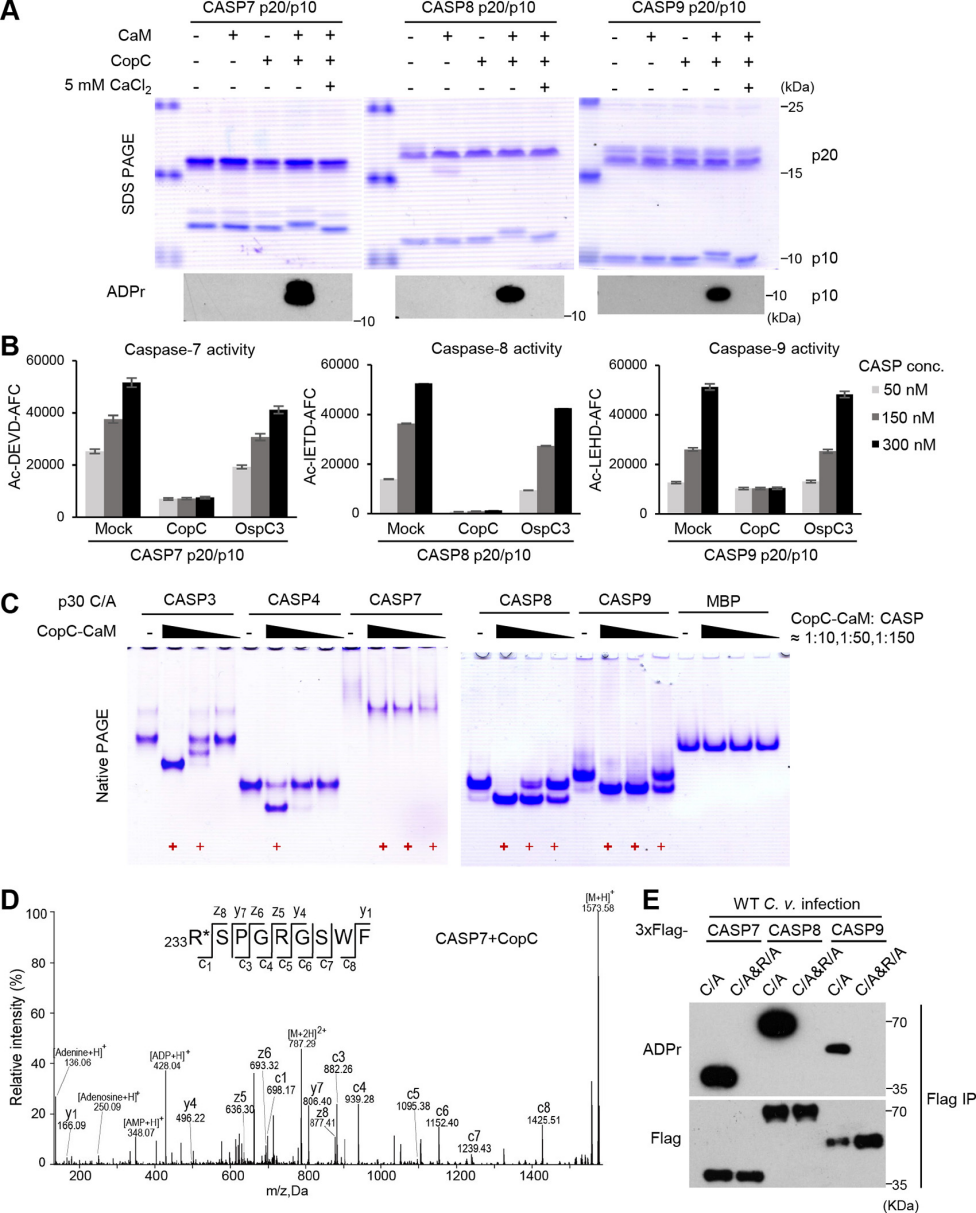
CaM binding activates CopC to ADP-riboxanate and inactivate caspase-7/8/9. (A and B) CaM-dependent ADP-riboxanation and inactivation of caspase-7/8/9 by CopC *in vitro*. The p20/p10 form of purified caspase-7/8/9 were reacted (30°C, 2 h) with CopC or CaM protein alone, or the CopC-CaM complex (with or without 5 mM CaCl_2_) at the molar ratio of 50:1. Proteins were separated on SDS-PAGE gels and assessed by Coomassie-blue staining and anti-ADPr immunoblotting (A). Indicated amounts of the caspases following the reaction were subjected to activity measurement using their specific fluorogenic peptide substrates. Data are shown as means ± SD from three determinations (B). (C) Native gel mobility-shift assay of caspase modification by the CaM-CopC complex. The p30 form of indicated caspases (C/A) or MBP was incubated with the CaM-CopC complex at the indicated molar ratio. Bold and normal red + symbol mark complete and partial modification of the caspase, respectively. (D) EThcD mass spectrometry analysis of CopC-modified caspase-7. The modified p10 subunit of caspase-7 in (A) was subjected to EThcD mass spectrometry analysis. Tandem mass spectrum of the modified peptide _233_RSPGRGSWF is shown. (E) Mutation of the arginine site in caspases blocks their ADP-riboxanation by CopC. 293T cells were transfected with 3xFlag-caspase-7/8/9 (C/A mutation alone or in combination with R233A in caspase-7, R413A in caspase-8, or R355A in caspase-9), and then infected with WT C. violaceum. The experiment was performed and analyzed similarly as those in Fig. 2A,

To verify the ADP-riboxanase activity of CopC and its specific modification of caspase-7/8/9, we purified the autoprocessed p20/p10 form of these caspases from E. coli. At the enzyme-substrate molar ratio of 1:50, purified CopC failed to ADP-riboxanate the three caspases. Notably, addition of CaM into the reaction rendered efficient ADP-riboxanation of the p10 subunits of caspase-7/8/9, evident from the complete mobility shift on the SDS-PAGE and detection by anti-ADPr immunoblotting (Fig. 3A). Importantly, CopC-modified caspase-7/8/9 was completely inactive in hydrolyzing their peptide substrates while incubation of the caspases with the OspC3-CaM complex showed no such effect (Fig. 3B). In line with the observation that CopC could not bind to calcium-loaded CaM, CaM-stimulated modification of caspase-7/8/9 by CopC was abrogated by 5 mM CaCl_2_ (Fig. 3A). We further observed that 100 μM calcium was sufficient to block CopC modification of caspase-7/8/9 (Fig. S4A). Notably, the modification was unaffected by 100 nM calcium, which is the resting concentration of calcium in host cytoplasm (Fig. S4A). We then measured cellular calcium concentration upon C. violaceum infection. The data showed that the cytoplasmic calcium concentration remained stable within 150 min after the infection despite an increase afterwards (likely due to the known cell lytic activity of C. violaceum [20]) (Fig. S4B). Treating the infected cells with Ca^2+^ ionophore Calcimycin but not Ca^2+^ chelator BAPTA-AM could counteract WT C. violaceum inhibition of apoptotic responses (Fig. S4C). Further, calcium-like peptide 3 (CALP3), an artificially designed peptide that targets the EF-hands of CaM to maintain CaM in its calcium-bound conformation (26), could also evidently attenuate apoptosis inhibition by C. violaceum (Fig. S4C). These data suggest that CopC can be activated by Ca^2+^-free CaM in infected host cells.

10.1128/mbio.00690-22.4 FIG S4 (A) In vitro reconstitution of caspase-9 (p20/p10) ADP-riboxanation by the CaM-CopC complex in the presence of different concentrations of CaCl_2_. The assay was performed similarly as that in Fig. 3A. (B) HeLa cells loaded with Fluo-4 AM were infected with WT C. violaceum. The fluorescence intensities were measured at the indicated time points postinfection. Calcium concentrations were calculated (see Materials and Methods) and the relative calcium concentrations (compared to uninfected cells) are shown. (C) HeLa cells were loaded with 10 μM BAPTA-AM (premixed with 0.04% Pluronic F-127 and added 1 h prior to infection), 2 μM Calcimycin, or 200 μM CALP3 and then infected with WT C. violaceum. One hour postinfection, cells were stimulated with 200 ng/mL TRAIL plus 100 μg/mLCHX for 90 min. The immunoblotting was performed similarly as that in Fig. 2B. (D) EThcD mass spectrometry analysis of CopC-modified caspase-8 (upper) and caspase-9 (lower). Tandem mass spectra of the modified caspase-8 peptide _413_RNPAEGTWY of CASP8 and caspase-9 peptide _355_RDPKSGSWY are shown. (E) Multiple sequence alignment of the p10 subunits of different caspases. Clustal W2 and ESPript 3.0 were used to derive and display the alignment, respectively. The conserved arginine modified by CopC is highlighted by a black rectangle. Download FIG S4, PDF file, 0.8 MB. Copyright © 2022 Liu et al. 2022 Liu et al. https://creativecommons.org/licenses/by/4.0/ This content is distributed under the terms of the Creative Commons Attribution 4.0 International license.

Similar to OspC3 modification of caspase-4/11, CopC-modified caspases also exhibited a remarkable shift on the native-PAGE gel. Taking advantage of this change, we compared the modification efficiency of different caspases by titrating the amounts of the CopC-CaM complex in the reaction (Fig. 3C). At the enzyme-substrate molar ration of approximately 1:10, caspase-3, -7, -8, and -9 all could be completely modified by CopC-CaM, and caspase-4 showed a partial modification while the irrelevant MBP protein was not modified. This suggests that excessive CopC lacks the substrate specificity toward the caspase-family members. Notably, when the relative amount of CopC-CaM complex versus the caspases was reduced to 1:150, efficient modification was only observed with caspase-7, -8, and -9, among which caspase-7 had a complete modification (Fig. 3C). Electron-transfer/higher-energy collision dissociation (EThcD)–mass spectrometry further revealed a 524-Dalton mass increase on Arg233, Arg413, and Arg355 in CopC-modified caspase-7, -8, and -9, respectively (Fig. 3D, S4D, and S4E). Supporting this observation, mutation of the arginine to alanine completely blocked C. violaceum infection-induced modification of caspase-7, -8 and -9 (Fig. 3E). Thus, CopC preferably ADP-riboxanates caspase-7/8/9 on a key arginine residue, which requires binding to CaM in host cells.

### Overall structure of CaM-CopC-CASP7 ternary complex.

To understand the mechanisms underlying CopC interaction with CaM and recognition of the caspase substrates, we tried to obtain the atomic structures of CopC in complex with CaM and the caspase. To aid structure determination, we deleted the N-terminal flexible region (50 residues) in CopC that bears the potential T3SS secretion signal. The resulting CopC ΔN50 retained comparable activities as full-length CopC in modifying caspase-7/8/9 (Fig. S5A). CopC ΔN50 alone was recalcitrant to be crystallized, but could form a high-quality stable complex with Ca^2+^-free CaM and caspase-7 p30 (C/A) (Fig. S5B). The gel-filtration elution profile of the ternary complex suggested a 2:2:2 stoichiometry, which was probably mediated by caspase-7 that by itself appeared as a dimer (Fig. S5B).

10.1128/mbio.00690-22.5 FIG S5 (A) Functional analyses of CopC N-terminal truncation mutants. The p20/p10 form of caspase-7, -8, or -9 were reacted with CopC (full-length [FL], ΔN50, or ΔN92) at the molar ration of 10:1 in the presence of CaM. (B) Gel-filtration chromatography analyses of CopC ΔN50, CaM. and caspase-7 p30 complex formation. The peak eluted from the column, the protein samples used for crystallization, as well as the crystal samples were assessed by Coomassie-stained SDS-PAGE gels. (C) A cartoon scheme of the 2:2:2 CaM-CopC-CASP7 complex structure. The dimeric ternary complex is mediated by caspase-7 molecules from two neighboring asymmetric units. The 2-fold crystallographic axis is shown. (D) Cartoon models of Ca^2+^-bound CaM alone (PDB code: 1CDL, left) and Ca^2+^-free CaM in complex with the IQ motif of myosin V (PDB code: 2IX7, right). Ca^2+^ are shown as green sphere. The middle shows CaM-C lobe in the ternary complex (magenta) overlaid with that in the Ca^2+^-bound CaM (upper) or Ca^2+^-free CaM (lower). (E) Dali search results of CopC-NTD structure. (F) Close-up view of the deduced NAD^+^-binding site in CopC-NTD and its position relative to the R233-containing loop of CASP7. Structures of isolated CASP7-p30 dimer (PDB code: 1K88) and CASP7-p20/p10 dimer (PDB code: 1K86) were superposed onto that of CASP7 in the ternary complex. The deduced NAD^+^-binding site in CopC-NTD is highlighted by a magenta dashed-line box with binding residues in stick models. Download FIG S5, PDF file, 1.1 MB. Copyright © 2022 Liu et al. 2022 Liu et al. https://creativecommons.org/licenses/by/4.0/ This content is distributed under the terms of the Creative Commons Attribution 4.0 International license.

**FIG 4:**
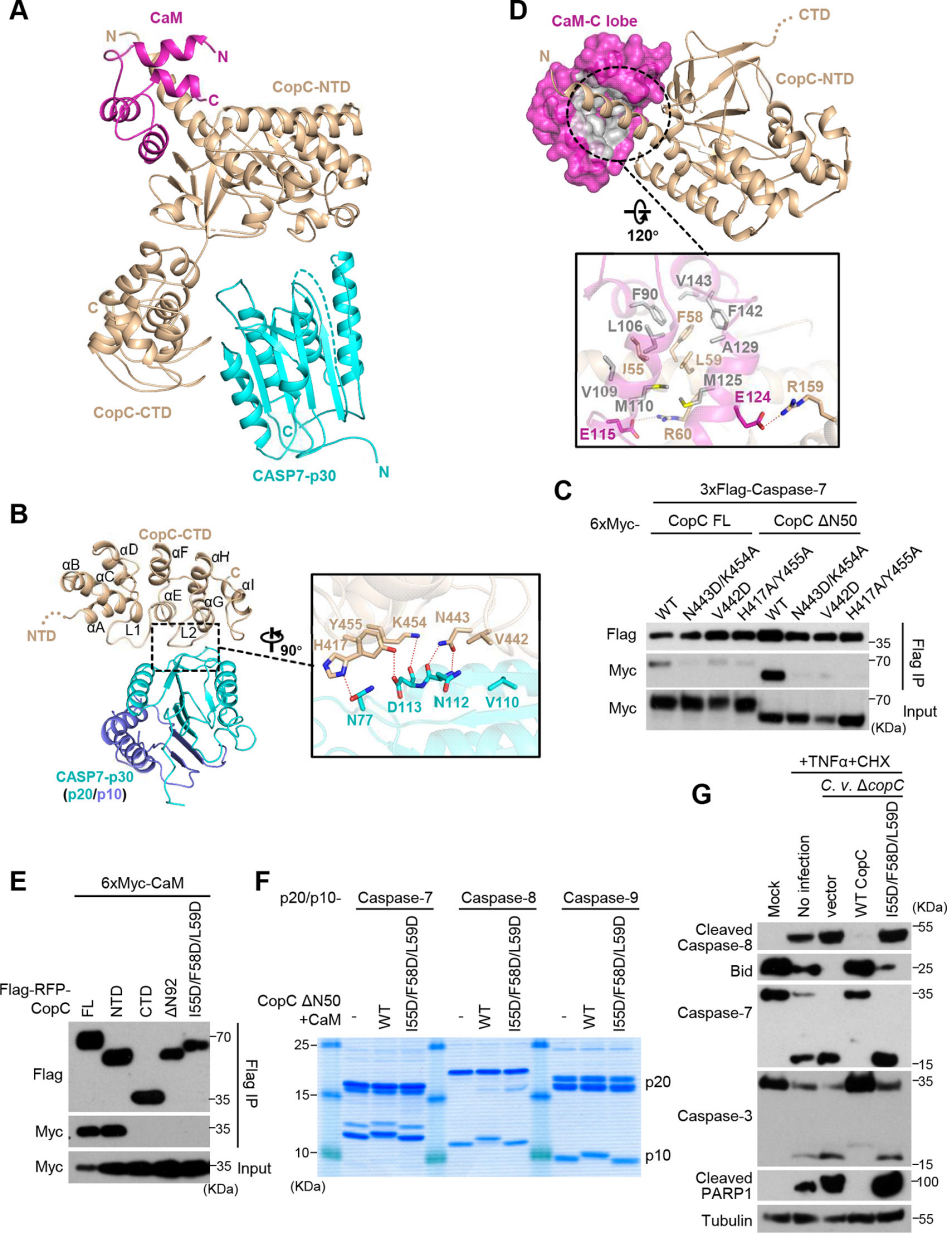
Crystal structure of the CaM-CopC-CASP7 complex. (A) Overall crystal structure of CopC ΔN50 in complex with CaM and caspase-7-p30 (C/A). Cartoon models for CopC ΔN50, CaM, and caspase-7 are colored in wheat, magenta, and cyan, respectively. (B) The interface between CopC-CTD and caspase-7. Secondary structures of CopC-CTD are labeled in order, in which L1 and L2 are the two loops that directly contact the p20 part of caspase-7. Close-up view of the intermolecular interface is shown on the right. Residues mediating CopC-CASP7 interaction are labeled and shown as sticks. Dotted lines are hydrogen bonds. (C) Mutagenesis analyses of CopC-CASP7 binding interface. 293T cells were cotransfected with 3xFlag-caspase-7 and 6xMyc-CopC (full-length [FL], ΔN50, or an indicated mutant). Cell lysates were subjected to anti-Flag immunoprecipitation followed by immunoblotting as shown. Immunoblotting of total cell lysates (Input) shows the expression of CopC. (D) The interface between CopC-NTD and CaM. CaM-C lobe is shown in surface presentation with the CopC-binding hydrophobic groove colored in gray. The lower is the closeup view of the interface. Residues mediating CaM-CopC interaction are labeled and shown as sticks. Dotted lines are hydrogen bonds. (E to G) Mutagenesis analyses of CaM-CopC binding interface. In E, 293T cells were co-transfected with 6xMyc-CaM and Flag-RFP-CopC (full-length [FL] or an indicated variant). Immunoprecipitation and immunoblotting were performed as those in C. In F, the p20/p10 form of caspase-7, -8 or -9 were reacted with CaM-CopC ΔN50 complex at the molar ration of 10:1, and then assessed by Coomassie-stained SDS-PAGE gel. In G, HeLa cells were infected with C. violaceum Δ*copC* complemented with an empty vector or CopC (WT or the I55DF58DL59D mutant); 1 h postinfection, cells were treated with 30 ng/mL TNF-α plus 100 μg/mL CHX. Cell lysates were blotted with indicated antibodies against core proteins in the apoptosis pathway. I55D/F58D/L59D are mutations in the CaM-binding surface in CopC-NTD.

The above ternary complex (referred to as CaM-CopC-CASP7 hereafter) was successfully crystallized, and the structure was determined to 3.6 Å in the space group *P4_3_2_1_2* by molecular replacement (Table 1). Each asymmetric unit in the structure contains one 1:1:1 ternary complex; two such complexes are related by a 2-fold axis that also relates the dimeric caspase-7 monomers, forming a 2:2:2 CaM-CopC-CASP7 complex (Fig. S5C). Given that each 1:1:1 complex is identical in the crystal lattice, structure of one representative complex in an asymmetric unit was further analyzed (Fig. 4A). In line with the two-domain architecture shared by OspC-family members, the overall structure of CopC takes a “V”-like shape, in which the N-terminal domain (NTD) and the C-terminal domain (CTD) act as the two arms. Caspase-7, situated at the open concave of the “V,” is accommodated by the two arms, but only contacts the CTD. A CaM molecule binds to the convex surface of CopC, acting as a small handle to support CopC-caspase-7 binding from the opposite side (Fig. 4A).

**TABLE 1:** Data collection and refinement statistics^*a*^

	CopC ΔN50-CaM-CASP7 p30^C186A^
Data collection	
Space group	*P4_3_2_1_2*
Cell dimensions	
*a, b, c* (Å)	90.87, 90.87, 426.11
*α, β, γ* (°)	90, 90, 90
Wavelength (Å)	0.97853
Resolution (Å)	47.65–3.60 (3.69-3.60)
*R_merge_* (%)	6.6 (112.0)
*I/σI*	18.46 (2.13)
Completeness (%)	99.9 (99.7)
Redundancy	8.3 (8.0)
Refinement	
Resolution (Å)	47.65–3.60
No. reflections	21794
*R_work_/R_free_* (%)^*b*^	27.43/31.37
No. atoms	
Protein	5515
B-factors (Å^2^)	
Protein	180.99
R.m.s deviations	
Bond lengths (Å)	0.001
Bond angles (°)	0.358
Ramachandran plot	
Favored (%)	92.90
Allowed (%)	6.52
Outlier (%)	0.58

a Values in parentheses are for the highest-resolution shell.

b 9.13% of reflections are randomly selected for calculating *R_free_*.

### Recognition of the caspase by CopC C-terminal ankyrin repeats.

Consistent with our previous analyses of OspC3, CopC-CTD is responsible for binding the substrate (caspase-7). The CaM-CopC-CASP7 structure shows that CopC-CTD (residues 345–487) adopts an all-α structure consisting of 9 amphiphilic helices, in which the central 6 helices (αC–αH) form 3 consecutive ankyrin repeats (Fig. 4B). Like the ankyrin repeats, the first two helices αA and αB also fold into an antiparallel helix pair, and the total of four helix pairs pack side by side to form a compact solenoid. The C-terminal helix αI acts as a cap, its hydrophobic face covering the outer side of the αG/αH pair. Connecting the three neighboring ankyrin repeats are two extended loops (L1 and L2), both of which take an ordered conformation and project outward from the convex surface of the solenoid with a ∼90° angle relative to the helix pairs. The hydrophilic faces of αE and αG, together with the turn region of L2, cooperatively constitute the major caspase-7-binding surface (Fig. 4B).

Caspase-7 in the ternary complex features a typical caspase fold, and its structure resembles that of isolated caspase-7 dimer determined previously, in which the p10 subunit mediates the dimerization (27). The R233-containing loop in the p10 stretches out of the caspase catalytic center and points to CopC-NTD (the enzymatic domain, see below), while the p20, specifically its outer face, directly interacts with CopC-CTD. N443 of the L2 loop and K454 at the N-terminus of αG in CopC form three hydrogen bonds with the mainchain of N112-D113 dipeptide located at the end of the outermost β-strand of caspase-7 p20 subunit. Y455 and H417 of CopC-CTD interact with the sidechains of D113 and N77 of caspase-7 through two additional hydrogen bonds. In addition to the hydrogen-bond network, V442 in the L2 loop of CopC-CTD is close to V110 from the outermost β-strand of caspase-7, supplying hydrophobic interactions between the enzyme and the substrate (Fig. 4B). Mutagenesis analyses of the binding interface showed that N443A/K454A, H417A/Y455A, and V442D mutations in CopC-CTD largely disrupted the co-immunoprecipitation interaction between full-length/ΔN50 CopC and caspase-7 (Fig. 4C). As CopC ΔN50 seemed be more dependent on the interface to bind caspase-7 (Fig. 4C), we cannot exclude the possibility that the N-terminal region in CopC may also affect substrate recognition.

### A distinct CaM-binding mode of CopC-NTD.

We have shown that CaM binding plays a critical role in activating CopC to ADP-riboxanate the substrate caspase. Previous studies have established structural understanding of CaM and CaM binding to its partner proteins. An intact CaM molecule is composed of two lobes (N- and C-lobe), each folding into a small 4-helix domain bearing two calcium-binding EF-hands. Saturated chelation of four Ca^2+^ by the EF-hands leads to a fully open conformation for each lobe, rendering the whole molecule a concave surface featuring an extended groove for partner binding (28). In the absence of calcium, the conformation of each lobe is alterable and the orientation between the two lobes is relatively flexible; consequently, the groove can recognize a distinct set of binding partners. IQ motif ([I,L,V]QxxxRGxxx[R,K]) is a characteristic element in many apo-CaM-binding proteins such as myosin V (Fig. S5D) (29). Our data show that only apo-CaM could bind and stimulate the activity of CopC. Interestingly, CopC does not possess the classic IQ motif, suggesting a uniqueness in CopC binding to apo-CaM.

In the CaM-CopC-CASP7 ternary structure, the C-lobe of CaM and its two EF-hands can be well modeled, whereas the N-lobe is omitted due to lack of interpretable electron densities (Fig. 4D). Given that the crystallized samples contained an intact CaM, the N-lobe is likely exposed to the solvent without stable interaction with CopC-NTD. The C-lobe of CaM binds α1 of CopC-NTD that protrudes from the turning point of the CopC convex surface. The C-lobe takes a semi-open conformation similarly as that in apo-CaM binding to the IQ motif in myosin V (Fig. S5D). A series of nonpolar residues located on the concave surface of the CaM C-lobe form a hydrophobic groove to grip the amphipathic α1 of CopC-NTD. The hydrophobic face of the N-terminal portion of α1 is composed of I55, F58, and L59, making extensive nonpolar interactions with CaM. Around the hydrophobic interactions, R60 and R159 of CopC-NTD also interact with E115 and E124 of the C-lobe through two hydrogen bonds, further strengthening the CopC-CaM binding (Fig. 4D).

Consistent with the above structural observations, CopC-NTD, but not CopC ΔN92, was sufficient for binding CaM in the co-immunoprecipitation assay (Fig. 4E and 1B). When the hydrophobic residues I55, F58, and L59 involved in CopC binding to CaM were mutated into aspartic acid, the resulting triple mutant CopC lost the ability to interact with CaM and failed to modify its substrate caspases despite the presence of CaM (Fig. 4E and F). During infection, CopC I55D/F58D/L59D, complemented into the C. violaceum Δ*copC*, also failed to block TNF-α-plus-CHX stimulation of the apoptotic program in HeLa cells, evident from the diminished cleavage of caspase-3, caspase-7, and PARP1 (Fig. 4G). These functional data validate the unique CaM-binding mode observed in the CaM-CopC-CASP7 ternary complex structure.

### CopC-NTD contains an ART-like catalytic center for ADP-riboxanation.

**FIG 5:**
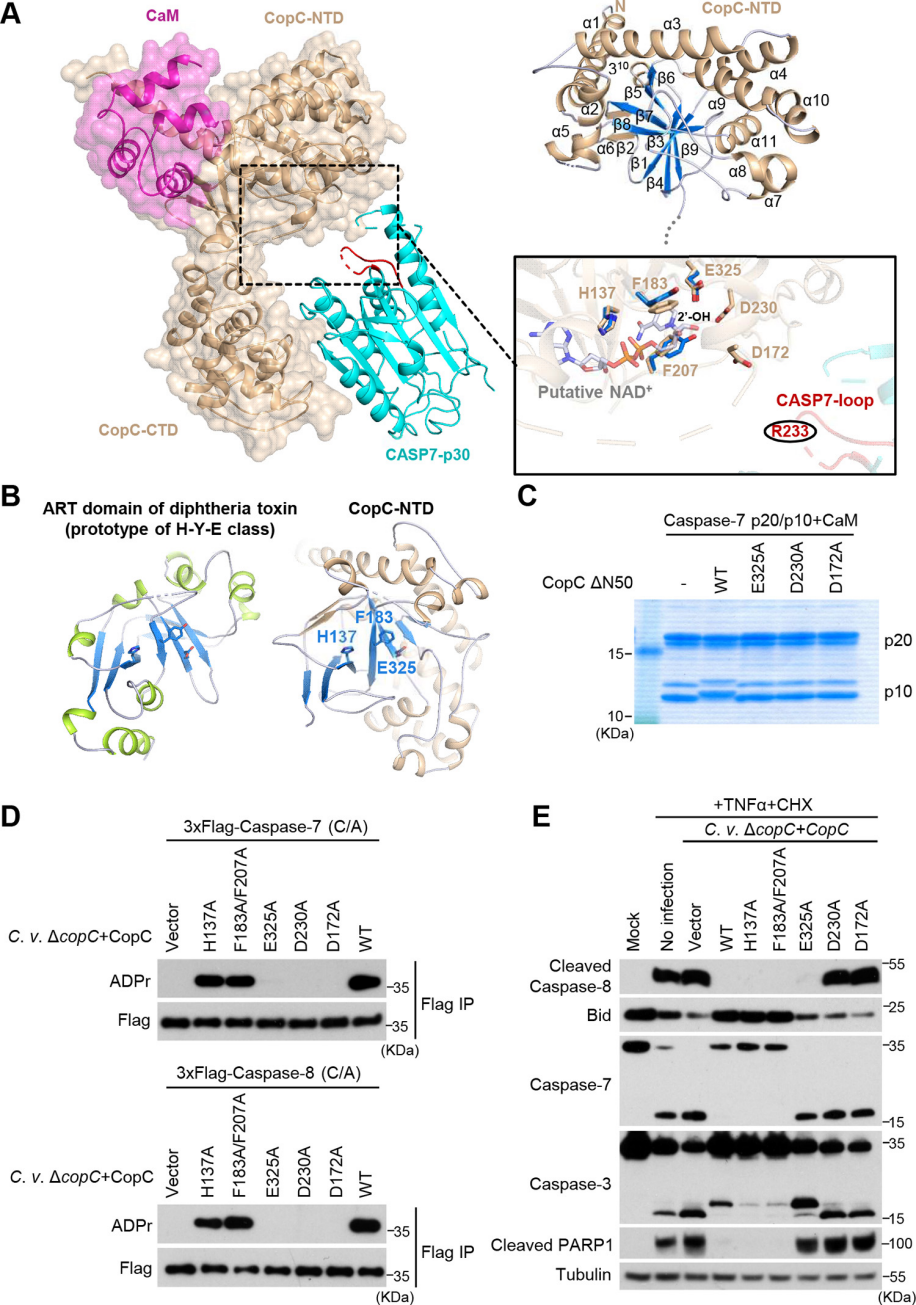
Structural insights into NAD^+^-dependent catalysis by CopC-NTD. (A) An NAD^+^-binding model deduced from CopC-NTD structure. Left, overall structure of CaM-CopC-CASP7 ternary complex with the potential NAD^+^-binding pocket highlighted by a dashed-line box. CaM and CopC are shown as cartoons overlaid with transparent surface map. CASP7 is shown as cartoons with the R233-containing loop colored in red. Upper right, cartoon model of CopC-NTD with numbered second structures. Lower right, cartoon model of CopC-NTD. The lower is a closeup view of the potential catalytic pocket in CopC-NTD. Residues crucial for NAD^+^ binding and ADP-riboxanation are shown as stick models and superimposed with the NAD^+^-bound structure of the H-Y-E triad in diphtheria toxin ART domain (PDB code: 1TOX). The modification site R233 in the caspase-7 loop is highlighted in red. (B) Cartoon schemes of diphtheria toxin ART domain and CopC-NTD. The central β-sheets that harbor the NAD^+^-binding triad are highlighted in blue, and the triad residues in both structures are shown as sticks. (C to E) Mutagenesis analyses of potential NAD^+^-binding and catalytic residues in CopC. In C, purified caspase-7 p20/10 proteins were subjected to modification by free CopC Δ50 or CaM-bound CopC Δ50 (WT or an indicated mutant). The reactions were then assessed by Coomassie-stained SDS-PAGE gel. In D, 293T cells expressing 3xFlag-caspase-7 or -8 (C/A mutants for both) were infected with C. violaceum Δ*copC* complemented with an empty vector or CopC (WT or an indicated point mutant). Lysates of infected cells were subjected to anti-Flag immunoprecipitation followed by immunoblotting as shown. In E, HeLa cells were infected with C. violaceum Δ*copC* complemented with an empty vector or CopC (WT or an indicated mutant); 1 h postinfection, cells were treated with 30 ng/mL TNF-α plus 100 μg/mL CHX. Cell lysates were blotted with indicated antibodies against core proteins in the apoptosis pathway.

The CopC-NTD (residues 51–344) adopts a globular domain that features a sandwich-like α/β Rossmann fold (Fig. 5A). The central core of CopC-NTD is a 7-stranded antiparallel β-sheet that can be divided into two subsheets (β1/β4/β9 and β3/β7/β5/β6), twisted by about 60°. α1-α2 at the N-terminus and α5–α6 in the linking region of β1-β3 flank one face of the central sheet. The long straight helix α3 crosses the outside of β6 toward the other face of the central sheet that is flanked by α4, α9-α10-α11, and α7-α8. A 2-stranded β-sheet (β2/β8) is situated at one end of the β3/β7 subsheet and assumes a perpendicular orientation relative to the β1/β4/β9 subsheet.

To understand the structural basis of the enzymatic activity in CopC-NTD, its structure was subjected to homology search using the Dali server (30). Z-scores of the top 10 hits were all below 4 (Fig. S5E), suggesting that CopC lacks overall structural similarity to known proteins. However, we noticed that many of the hits shared the ADP-ribosyltransferase (ART) fold featuring a NAD^+^-binding Rossmann fold. ART proteins have been classified into two major classes based on their catalytic triads (31). One is called diphtheria toxin-like ART featuring an H-Y-E (His-Tyr-Glu) catalytic motif; the other is cholera toxin-like ART sharing an R-S-E (Arg-Ser-Glu) motif. Close examination of CopC-NTD structure revealed a notable resemblance between its Rossmann fold and that of diphtheria toxin, but unlike diphtheria toxin, CopC-NTD bears an H-F-E (His137-Phe183-Glu325) rather than H-Y-E motif (Fig. 5B). When the H-Y-E triad of diphtheria toxin was overlaid onto the H-F-E motif of CopC-NTD, the NAD^+^ ligand from diphtheria toxin was well accommodated by the potential NAD^+^-binding sites in CopC (Fig. 5A). In this deduced model, an additional aromatic residue Phe207, in addition to Phe183, stacks with the nicotinamide group via π-π interactions to further support the NAD^+^ binding. Glu325 of the catalytic triad of CopC-NTD was found to form a strong hydrogen bond with the 2’-hydroxyl group (2’-OH) of nicotinamide-linked ribose (N-ribose), facilitating the leaving of nicotinamide following transfer of the ADP-ribose moiety. We then examined the functional relevance of these potential catalytic-site residues. The single E325A mutation in the deduced H-F-E triad of CopC completely abolished ADP-riboxanation of caspase-7/8 *in vitro* and during C. violaceum infection (Fig. 5C and D). The mutant protein failed to inhibit signal-induced activation of the apoptotic program in C. violaceum-infected HeLa cells (Fig. 5E). However, other mutations H137A and F183A/F207A in the potential catalytic triad did not show an apparent inhibition of CopC modification of caspase-7/8 and exhibited little effects on apoptotic program during C. violaceum infection (Fig. 5D). These suggest that H137 and F183/F207 may not be the key residues for chelating NAD^+^ as suggested by the modeled structure, or that their role can be compensated by other unidentified residues.

According to our model, the NAD^+^-binding region in CopC-NTD is located on the concave surface of the “V”-shape structure, facing the arginine acceptor (R233)-bearing loop in caspase-7. Unfortunately, the loop (residues 230–238 of caspase-7) was not modeled due to the lack of high-quality electron densities. When overlapping the structures of unprocessed caspase-7-p30 and processed caspase-7-p20/p10 dimer in isolation with the structure of caspase-7 in the ternary complex, the R233-containing loop, assuming a fixed conformation in isolated caspase-7 structures, had no direct contacts or clashes with CopC-NTD including its catalytic pocket (Fig. S5F). Thus, CopC shall undergo the same extents of domain movement so that caspase-7, upon recruitment to the enzyme, can present its R233 to the catalytic pocket of CopC for modification. We then performed mutagenesis analyses of residues in CopC whose structural positions predict an engagement into NAD^+^-dependent modification of R233. The analyses revealed that alanine substitution of D230 or D172 in CopC-NTD abolished the ADP-riboxanation of caspase-7/8 *in vitro* and during C. violaceum infection (Fig. 5C and D). Consistently, C. violaceum Δ*copC* harboring CopC D230A or D172A failed to inhibit the apoptotic pathway in HeLa cells (Fig. 5E). We speculate that the two acidic residues might be involved in activating the arginine acceptor and 2’-OH of N-ribose (Fig. 5A). At this stage, the exact mechanism underlying site-specific arginine ADP-riboxanation by CopC requires further investigations by obtaining structures bound with both the NAD^+^ ligand and the caspase as well as structures of catalytical intermediates.

## DISCUSSION

The OspC family is widely present in Gram-negative bacteria, including *Shigella*, *Vibrio*, Salmonella, *Erwinia*, and *Chromobacterium*. Here, we report the molecular mechanism of another OspC-family member, the CopC effector from C. violaceum. We identify two groups of host proteins that can bind to CopC in mammalian cells. One is EF-hand proteins (32), including CaM, CaM-like protein 3, and Troponin C that share similar biochemical/structural properties. We show that calcium-free CaM can activate or greatly stimulate the ADP-riboxanase activity of CopC. Activation of bacterial effectors/toxins by CaM binding has been reported in other cross-kingdom scenarios. CyaA from Bordetella pertussis and edema factor from Bacillus anthracis, both of which function to elevate the cyclic AMP level in host cells, are switched on by CaM binding (33–35); SidJ, a Legionella pneumophila type IV secretion system effector, catalyzes glutamylation of SidE-family effectors from the same bacteria, and its activity also relies on binding to CaM (36–38). Ectopic expression of CaM in L. pneumophila prematurely provokes the action of SidJ to modify SidE-family proteins, phenocoping mutant bacteria lacking the SidE family (39). However, in the absence of genuine substrates (caspases) of CopC, CaM expression in C. violaceum cannot reduce the cellular contents of NAD^+^ and affect bacterial growth. Unlike most eukaryotic CaM-regulated proteins that selectively bind calcium-loaded CaM, CopC exploits the calcium-free CaM abundantly present in host cytoplasm. Crystal structure of the CaM-CopC-CASP7 ternary complex reveals that CopC uses its NTD to bind CaM, which is different from host CaM-regulated proteins that often use an IQ motif.

The other group of CopC-binding proteins are caspase-3 and -7. This suggests that CopC, unlike OspC3, may target apoptotic caspases and block apoptosis. CopC has intrinsically high enzymatic activity and can modify multiple caspases when its amount is excessive in the artificial system. In C. violaceum infection, CopC efficiently modifies caspase-7, -8, and -9, but not caspase-3, -4, or -6. C. violaceum, but not its Δ*copC* strain, potently blocks apoptotic cascade at the caspase-8/9 steps. Caspase-3 is most close to caspase-7, and both caspases bind CopC comparably, stronger than caspase-8/9. However, caspase-3 is a poor ADP-riboxanation substrate of CopC. Analyses of the structural interface between caspase-7 and CopC fail to give mechanistic insights into CopC substrate selectivity. Apparently, the substrate-binding affinity and the modification efficiency of CopC do not correlate well for different caspases, likely due to the sophisticated and dynamic process during CopC ADP-riboxanation of its substrates. How CopC coordinates substrate selectivity and catalytic efficiency awaits further biochemical or biophysical investigations.

Our structural analyses reveal an ART-like catalytic center in CopC-NTD, responsible for its ADP-riboxanation activity. Like other ARTs, CopC relies on a conserved glutamic acid (Glu325) in its catalytic triad to mediate catalysis. We also identify two aspartate residues around the catalytic center that are crucial for catalyzing arginine ADP-riboxanation, suggesting a new catalytic scheme employed by CopC. Due to the lack of high-quality electron densities for the arginine-acceptor region in the CaM-CopC-CASP7 structure and the lack of catalytic intermediate structure, we are unable to depict a full enzymatic mechanism for CopC-catalyzed ADP-riboxanation. Despite this, our study does unveil a new mode of CaM-regulated activity in CopC that effectively hijacks the apoptotic program in mammalian cells.

In addition to S. flexneri, C. violaceum is another bacterial pathogen that bears a functional ADP-riboxanase effector. S. flexneri is a human pathogen and causes bacillary dysentery while C. violaceum infection in humans is opportunistic. Besides OspC3 that could ADP-riboxanate and inactivate caspase-4 to block cytosolic LPS-induced pyroptosis (9), S. flexneri also harbors OspC1 reported to inhibit caspase-8 (40) presumably through the same ADP-riboxanation, as well as an OspD3 effector that can degrade RIPK1 and RIPK3 to block necroptosis (41). Thus, S. flexneri not only hijacks host NF-κB and MAPK pathways (42–44), but also suppresses multiple host cell death processes, which allows the bacteria to escape from the inflammatory insults and establish the infection in the host. Different from OspCs, C. violaceum-derived CopC is inefficient in suppressing pyroptosis. Despite that C. violaceum has evolved to inhibit the apoptosis and NF-κB pathways, its infection features extensive lytic death through NLRC4 inflammasome-mediated pyroptosis as well as the action of secreted hemolytic toxins. Lytic cell death, particularly pyroptosis, is highly pro-inflammatory, which shall magnify C. violaceum-stimulated immune defenses including neutrophil-mediated killing activity (20, 45–47). This may partially explain why C. violaceum is less virulent and has a lower probability of causing severe diseases in immunocompetent humans.

## MATERIALS AND METHODS

### Plasmids, antibodies, and reagents.

The coding region of *copC*, *ospC1*, *ospC2*, and *ospC3* were PCR-amplified from the genomic DNA of C. violaceum and S. flexneri. The coding region of human *CASP*s, *CALM1*, *GSDMD*, and mouse *Casp11* were from the Life Technologies Ultimate ORF collection. CopC, OspC3, and CaM were cloned into a broad-host-range vector pBBR1 for expression in C. violaceum. 6xMyc-, 3xFlag- or Flag-RFP-tagged CopC, OspC, CaM, and CASPs were cloned into pCS2 vector for transient expression in mammalian cells. For expression in E. coli, CopC was constructed into the pGEX-6P-2 vector. CaM was cloned into pSUMO with N-terminal tandem His and SUMO tags. CASPs-p30 WT or CA mutants were inserted into pET21a vector with a C-terminal His tag. CASP4, GSDMD, and FKBP-tagged CASP8/9-p30 were inserted into FUIPW or PWPI plasmid for stable expression in HeLa cells. tBid was cloned into a modified pLenti-NIrD vector for inducible expression in HeLa cells. Truncations or point mutations were generated by standard PCR strategy or QuikChange site-directed mutagenesis. All plasmids were verified by DNA sequencing.

Antibodies for Tubulin (T5168) and Flag (F7425) were from Sigma-Aldrich. Antibodies for poly/mono-ADP ribose (E6F6A), caspase-8 (1C12), cleaved caspase-8 D374 (18C8), caspase-7 (D2Q3L), caspase-3 (#9662), cleaved PARP D214 (D64E10), and human Bid (#2002) were rom Cell Signaling Technology. Anti-GSDMD (ab209845), anti-caspase9 (5B4), and anti-Calmodulin 1/2/3 (ab45689) antibodies were purchased from Abcam and MBL Life Science, respectively. Antibody for Myc tag (HX1821) was obtained from Huaxingbio.

Tenascin-C (TNC)-Fcγ-FasL protein was provided by Jianhua Sui (National Institute of Biological Sciences, Beijing). Recombinant human TNF-α (RTNFAI), Fluo-4 AM (F14201), and BAPTA-AM (B6769) were from Invitrogen. Cycloheximide (HY-12320), recombinant TRAIL (1121-TL), ATP (FLAAS), anti-Flag M2 gel (F2416), β-NAD (N0632), protease inhibitor cocktail (11697498001), AP20187 (SML2838), LPS (L4130), perchloric acid 70% (244252) and doxycycline (24390) were from Sigma-Aldrich. Ac-DEVD-AFC (14459), Ac-IETD-AFC (17480), and Ac-LEHD-AFC (17051) were from Cayman Chemical. CALP3 (HY-P1075) and Calcimycin (HY-N6687) were from MedChemExpress. Glutathione-Sepharose beads (17513202) and Ni-NTA agarose resin (17057502) were purchased from GE Healthcare. Cell culture products were from Life Technologies; all other reagents were Sigma-Aldrich products unless noted.

### Cell culture, transfection, and apoptosis induction.

HeLa and 293T cells were obtained from ATCC and cultured in Dulbecco’s modified Eagle’s medium (DMEM) supplemented with 10% FBS and 2 mM l-glutamine at 37°C in a 5% CO_2_ incubator. JetPRIME (Polyplus Transfection) was used for transient transfection following the manufacturer’s instructions. For stable expression, lentiviral constructs were transfected into 293T cells together with the packing plasmids psPAX2 and pMD2G. The supernatants were collected 48 h after transfection and used to infect HeLa cells for another 48 h. Stable expression cells were sorted by flow cytometry or selected by 5 μg/mL puromycin (InvivoGen).

Electroporation of 0.2 μg of LPS and indicated amounts of recombinant proteins into HeLa cells were carried out using the NeonTransfection System (ThermoFisher Scientific) following the manufacturer’s instructions with the following parameters: 1480 V, 10 ms, 2 pulses. Cell viability was measured 2 h after electroporation and determined by the CellTiter-Glo Luminescent Cell Viability Assay (Promega). To induce apoptosis, HeLa cells were treated with 1 μg/mL TNC-Fcγ-FasL or 30 ng/mL TNF-α in combination with 100 μg/mL CHX for 2 h, or 200 ng/mL TRAIL plus 100 μg/mL CHX for 1.5 h.

### Bacterial culture and infection.

C. violaceum 12472 and S. flexneri 2a 2457T were used in cell infection assay. C. violaceum Δ*copC* and S. flexneri Δ*ospC3* were generated previously (9). pBBR1-CopC or OspC3 plasmid was electroporated into the bacteria using Gene Pulser Xcell Electroporation Systems (Bio-Rad) following the manufacturer’s instructions. HeLa cells were seeded onto glass coverslips in 24-well plates (1 × 10^5^ cells/well) and cultured for 24 h at 37°C in a 5% CO_2_ incubator. For infection, C. violaceum was grown overnight in Tryptic Soy Broth (TSB) medium containing 0.004% (wt/vol) Congo red (Sigma) at 37°C and then subcultured (1:33) in fresh TSB-Congo red medium for 3.5 h. S. flexneri Δ*ospC3* was grown in 2×YT medium at 37°C overnight and then subcultured (1:33) in fresh 2×YT medium for 5 h. Bacteria culture was then added onto cells (MOI: 100) with centrifugation (800 *g*, 5 min). After 1-h incubation at 37°C, cells were washed with phosphate-buffered saline (PBS) twice and incubated in fresh DMEM containing 100 μg/mL gentamicin for further infection.

To examine the uptake of C. violaceum, HeLa cells (90% confluence) in 12-well plates were infected with C. violaceum WT, Δ*copC*, Δ*copC* harboring pBBR1-CopC, or Δ*civC* at the MOI of 100 with centrifugation (800 *g*, 5 min). After 1-h incubation at 37°C, cells were washed three times with PBS and then lysed in 1 mL of cold PBS containing 0.1% TritonX-100. C. violaceum CFU were determined by serial-dilution plating on TSB plates.

### Recombinant protein expression and purification.

For recombinant expression and purification, pGEX-6P-2-CopC ΔN50, pET21a-caspase-7 (ΔN23), and pSUMO-CaM were transformed into E. coli BL21(DE3) and protein expression was induced with 0.2 mM isopropyl-β-d-thiogalactopyranoside (IPTG) at 18°C overnight in LB medium containing appropriate antibiotics. CopC was affinity-purified using glutathione-Sepharose beads in the buffer containing 20 mM MES (pH 6.0), 150 mM NaCl, and 5 mM dithiothreitol (DTT). The GST tag was removed by overnight digestion with homemade PreScission protease at 4°C. Caspase-7 and CaM were both purified by Ni-Sepharose affinity chromatography in the buffer containing 20 mM Tris-HCl (pH 8.0), 150 mM NaCl, 20 mM imidazole, 10 mM β-mercaptoethanol, and 5% (vol/vol) glycerol. CaM was obtained from the flow-through fraction after removal of the SUMO tag by overnight digestion with homemade Ulp1 protease at 4°C. To prepare CaM-CopC-CASP7 ternary complex, the three proteins were mixed together at the molar ratio of 1:1:1 and incubated overnight at 4°C. The complex was purified by Superdex G200 gel-filtration chromatography, concentrated and stored at −80°C in the buffer containing 20 mM Tris-HCl (pH 8.0), 150 mM NaCl, and 5 mM dithiothreitol (DTT).

### Crystallization, data collection, and structure determination.

Crystallization was carried out at 18°C using the sitting-drop vapor diffusion method by mixing 1 μL of the CaM-CopC-CASP7 ternary complex solution and 1 μL of reservoir solution in the presence of 2 mM NADH. Initial crystals were observed in several conditions in the Index kit (Hampton Research). Qualified crystals appeared within a week in the reservoir buffer containing 100 mM Bis-Tris propane (pH 7.5), 1.5 M lithium sulfate; the crystals were transferred into a cryoprotectant solution containing the reservoir buffer supplemented with 15% glycerol before flash-freezing with liquid nitrogen. Diffraction data were collected at the Shanghai Synchrotron Radiation Facility (Shanghai, China) beamline BL19U1 and processed with XDS package (48). Initial phase was determined by molecular replacement using the caspase-7 structure (PDB code: 1K88) (27) and a CopC structure predicted by AlphaFold (49) as starting models in the PHENIX suite (50). The structure was refined with PHENIX, and manual modeling was performed between refinement cycles in Coot (51). Data collection and refinement statistics are summarized in Table 1. Quality of the final model was validated by MolProbity (52). All structural figures were prepared in PyMOL.

### Immunoprecipitation and cell imaging.

To identify CopC-interacting proteins, three 10-cm dishes of 293T cells were transiently transfected with Flag-RFP or Flag-RFP-CopC plasmid. Twenty-four h later, cells were lysed in the buffer containing 50 mM Tris-HCl (pH 7.6), 150 mM NaCl, 2 mM EDTA, 1% Triton X-100, and a protease inhibitor cocktail. The soluble fraction was obtained by centrifugation at 21,000 *g* for 10 min at 4°C. Prewashed anti-Flag M2 affinity gel was added and incubated for 2 h at 4°C with constant rotation. The immunoprecipitates were washed five times with the lysis buffer, eluted with Flag peptide (0.5 mg/mL), and then denatured by the SDS loading buffer at 95°C for 10 min. The samples were loaded onto an SDS-PAGE gel for mass spectrometry.

To detect the interaction between CopC and CaM (or caspases), 293T cells were transiently transfected with indicated plasmids. Cells were lysed in the lysis buffer and anti-Flag immunoprecipitation was performed. To assay the effect of calcium on CopC-CaM interaction, cells were lysed in a buffer containing 50 mM Tris-HCl (pH 7.6), 150 mM NaCl, 5 mM CaCl_2_, 1% Triton X-100, and the protease inhibitor cocktail. After incubation with the anti-Flag M2 resin, the immunoprecipitates were washed with the same buffer and then boiled with the SDS loading buffer for 10 min. Samples were loaded onto a 4%–20% SDS-PAGE gel followed by immunoblotting. For immunofluorescence microscopy, cells were fixed with 4% paraformaldehyde for 20 min at room temperature (RT) and washed twice with PBS. Cell nuclei were stained with DAPI for 10 min at RT. Images were taken on the Zeiss LSM 800 confocal laser scanning microscope.

### Mass spectrometry.

Protein bands on the SDS-PAGE gel were destained and in-gel digested with sequencing grade chymotrypsin overnight at 37°C. Peptides were extracted with 5% formic acid/50% acetonitrile and 0.1% formic acid/75% acetonitrile sequentially and then concentrated to 20 μL. To detect ADP-riboxanation modification, the extracted peptides were separated on an analytical capillary column (100 μm 15 cm) packed with 3 μm spherical C18 reversed phase material (Dr. Maisch GmbH, Germany). An EASY-nLC 1200 (ThermoFisher Scientific) was used to generate the following HPLC gradient: 0–35% B in 35 min, 35–98% B in 12 min (A = 0.1% formic acid and 99.9% H_2_O; B = 80% acetonitrile, 0.1% formic acid and 20% H_2_O). The eluted peptides were sprayed into an ORBITRAP Fusion Lumos mass spectrometer (ThermoFisher Scientific) equipped with a nano-ESI ion source. The mass spectrometer was operated in data-dependent mode with one MS scan followed by EThcD MS/MS scans for each cycle. Database searches were performed on an in-house Mascot server (Matrix Science Ltd.) against caspase-4, -7, -8, and -9 protein sequences. The search parameters were the following: 10 ppm mass tolerance for precursor ions; 0.02 Da mass tolerance for product ions; three missed cleavage sites were allowed for chymotrypsin digestion. The following variable modifications were included: oxidation on methionine, carbamidomethylation on cysteine, ADP-riboxanation on arginine. The search results were filtered with both peptide significance threshold and expectation value to be below 0.05. The tandem mass spectra of matched ADP-riboxanated peptides were manually checked for their validity.

To identify CopC-binding proteins, extracted peptides were separated by an analytical capillary column (50 μm, 10 cm) packed with 5 μm spherical C18 reversed-phase material (YMC). A Waters nanoAcquity UPLC system was used to generate the following HPLC gradient: 0–30% B in 60 min, 30–70% B in 15 min (A = 0.1% formic acid in water; B = 0.1% formic acid in acetonitrile). The eluted peptides were sprayed into a LTQ ORBITRAP Velos mass spectrometer (ThermoFisher Scientific) equipped with a nano-ESI ion source. The mass spectrometer was operated in data-dependent mode with one MS scan followed by 10 HCD (High-energy Collisional Dissociation) MS/MS scans for each cycle. Database searches were performed against the IPI (International Protein Index) human protein database. The search parameters were the following: 7 ppm mass tolerance for precursor ions; 0.02 Da mass tolerance for product ions; two missed cleavage sites were allowed for trypsin digestion. Methionine oxidation was set as variable modification. The search results were filtered with both peptide significance threshold and expectation value to be below 0.05.

### NAD^+^ hydrolysis assay.

A fluorescence-based NAD^+^ hydrolysis assay was performed as previously described with modifications (53). Briefly, recombinant CopC, CaM, or CopC-CaM proteins were incubated with 400 μM NAD^+^ in a buffer containing 20 mM Tris-HCl (pH 7.6), 75 mM NaCl, and 2 mM MgCl_2_, without or without 5 mM CaCl_2_. Reactions were conducted at 16°C for 5 h and terminated by 2 M NaOH (final concentration) and incubated in the dark at RT for 30 min. Samples were transferred to a 96-well white solid plate (Costar) and analyzed using the Spark multimode microplate reader (Tecan) at excitation and emission wavelengths of 360 nm and 420 nm, respectively. NAD^+^ amount in each reaction was determined by interpolation from a standard curve.

### *In vitro* reconstitution of ADP-riboxanation.

The reaction was carried out in a buffer containing 50 mM Tris-HCl (pH 7.6), 150 mM NaCl, 2 mM DTT, and 200 μM NAD^+^. CaCl_2_ at different concentrations was added into the reaction when required. Caspase proteins, CopC, and CaM were incubated at the indicated molar ratio at 30°C for 2 h. Samples of the *in vitro* reaction were loaded and separated on a native-PAGE gel in a buffer containing 25 mM Tris (pH 8.3) and 192 mM glycine or an SDS-PAGE gel followed by staining with Coomassie brilliant blue R-250 or mass spectrometry analyses.

### Caspase activity measurement.

To measure caspase activity in C. violaceum infected cells, cells were lysed with the lysis buffer containing 20 mM PIPES (pH 7.4), 150 mM NaCl, and 0.5% CHAPS for 15 min. Protein concentrations were normalized using a bicinchoninic acid (BCA) kit (Thermo Scientific). Cell lysates containing equal amounts of proteins or indicated amounts of purified caspase were added to the reaction buffer containing 20 mM PIPES (pH 7.4), 150 mM NaCl, 5% sucrose, 0.1% CHAPS, 10 mM DTT, and 100 μM indicated fluorogenic substrates. The reactions were performed for 1 h at 37°C in the dark. Caspase activities were quantified by detecting the fluorescence of free AFC.

### Calcium and NAD^+^ measurements.

To measure cellular calcium concentration during infection, HeLa cells were digested with trypsin and washed twice with PBS. Digested cells were resuspended to a final concentration of 10^6^/mL in HBSS (Hanks balanced salt solution) containing 20 mM HEPES, 0.1% BSA, 2 μM Fluo-4 AM (Invitrogen), and 0.04% Pluronic F-127. After 40-min incubation, cells were washed twice with PBS and incubated in HBSS containing 20 mM HEPES, 0.1% BSA, and 1.5 mM CaCl_2_ for another 20 min at RT. Cells were then seeded on 96-well glass-bottom microplates (Perkin Elmer) for further treatment. Fluorescence intensity was analyzed using the Spark multimode microplate reader (Tecan) at excitation and emission wavelengths of 485 nm and 535 nm, respectively. Calcium concentration was calculated as follows: [Ca^2+^] = K_d_ (F–F_min_)/(F_max_–F). The K_d_ of Fluo-4 AM is 345 nM; F is the fluorescence signal intensity; F_max_ is the fluorescence signal intensity measured in the presence of 0.1% Triton X-100; F_min_ is the fluorescence signal intensity measured under 5 mM EGTA and 0.1% Triton X-100 in HBSS containing 20 mM HEPES.

To measure NAD^+^ concentration in C. violaceum, colonies of bacteria harboring pBBR1 or pBBR1-CaM were grown overnight, transferred 1:100 to fresh media, and further grown at 37°C until the absorbance (OD_600_) reached 0.8. We harvested 2.5 mL of bacteria culture by centrifugation. Metabolites from bacterial pellets were extracted using 200 μL of 10% perchloric acid, and then placed on ice for 20 min prior to centrifugation. The supernatants were transferred to a clean tube and one-third volume of 3 M K_2_CO_3_ was added. NAD^+^ was measured by HPLC-MS. A series of NAD^+^ standard solutions (5, 10, 20, 50, 100, 200, 500, 1,000, 2,000, 5,000, and 10,000 nM) were prepared by serial dilution of 1 mM stock with 50% methanol. Standard solutions and the above biological extracts were transferred to 250-μL inserts in auto-sampler vials. Analyses were performed on an Agilent 1290 infinity UHPLC system coupled to an Agilent 6495 triple quadrupole mass spectrometer with ESI source. A Poroshell 120 HILIC-Z column (2.1 mm × 100 mm, 2.7 μm) was used for separation. The injection volume was 2 μL. The mobile phases were 25 mM ammonium acetate and 25 mM ammonium hydroxide in water (A) and acetonitrile (B). The following gradient was applied: 0–6 min, 80–60% B; 6–7 min, 60% B; 7–7.5 min, 60–80% B; 7.5–10 min, 80% B. The flow rate was 0.4 mL/min, and column temperature was 35°C. Mass spectrometer was operated in multiple reaction monitoring (MRM) mode at unit resolution in positive mode, and optimized MRM transition of *m/z* 664.12→135.9 (CE 15V) was applied to analyze NAD^+^. The dwell time was 200 ms. The results were processed using Agilent Mass Hunter Quantitative Analysis B.07.00 software.

### Data availability.

The crystal structure of CaM-CopC-CASP7 ternary complex was deposited in the Protein Data Bank (PDB) under the accession number 7WZS.
