# Supplementary material for: Calmodulin Binding Activates Chromobacterium CopC Effector to ADP-Riboxanate Host Apoptotic Caspases
Source: mBio. 2022 Apr 21;13(3):e00690-22. doi: 10.1128/mbio.00690-22 (PMC9239266; doi:10.1128/mbio.00690-22)
Supplement: FIG S1 [file mbio.00690-22-sf001.pdf]

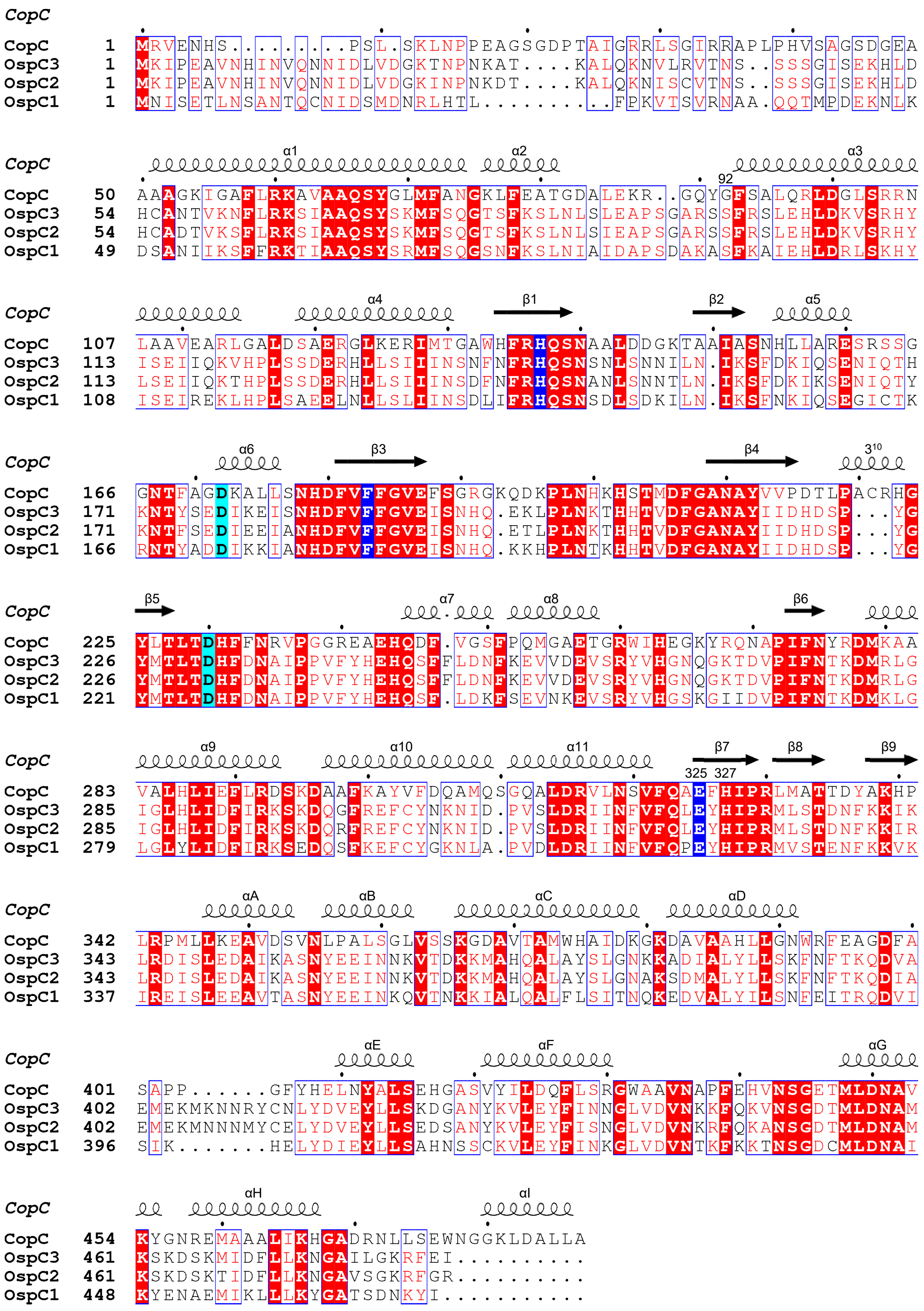


**FIG S1** Sequence alignment of *C. violaceum* effector CopC, *S. flexneri* effectors OspC1, OspC2, and OspC3. ClustalW2 was used to derive the alignment displayed using ESPript 3.0. Identical residues are highlighted by dark red background and conserved residues are in red. Secondary structures of CopC are labeled and shown on top of the alignment. The putative $NAD^{+}$-binding H-F-E triad and two catalytical residues (D172/D230) are highlighted by blue and cyan background, respectively.
