# Supplementary material for: Calmodulin Binding Activates Chromobacterium CopC Effector to ADP-Riboxanate Host Apoptotic Caspases
Source: mBio. 2022 Apr 21;13(3):e00690-22. doi: 10.1128/mbio.00690-22 (PMC9239266; doi:10.1128/mbio.00690-22)
Supplement: FIG S2 [file mbio.00690-22-sf002.pdf]

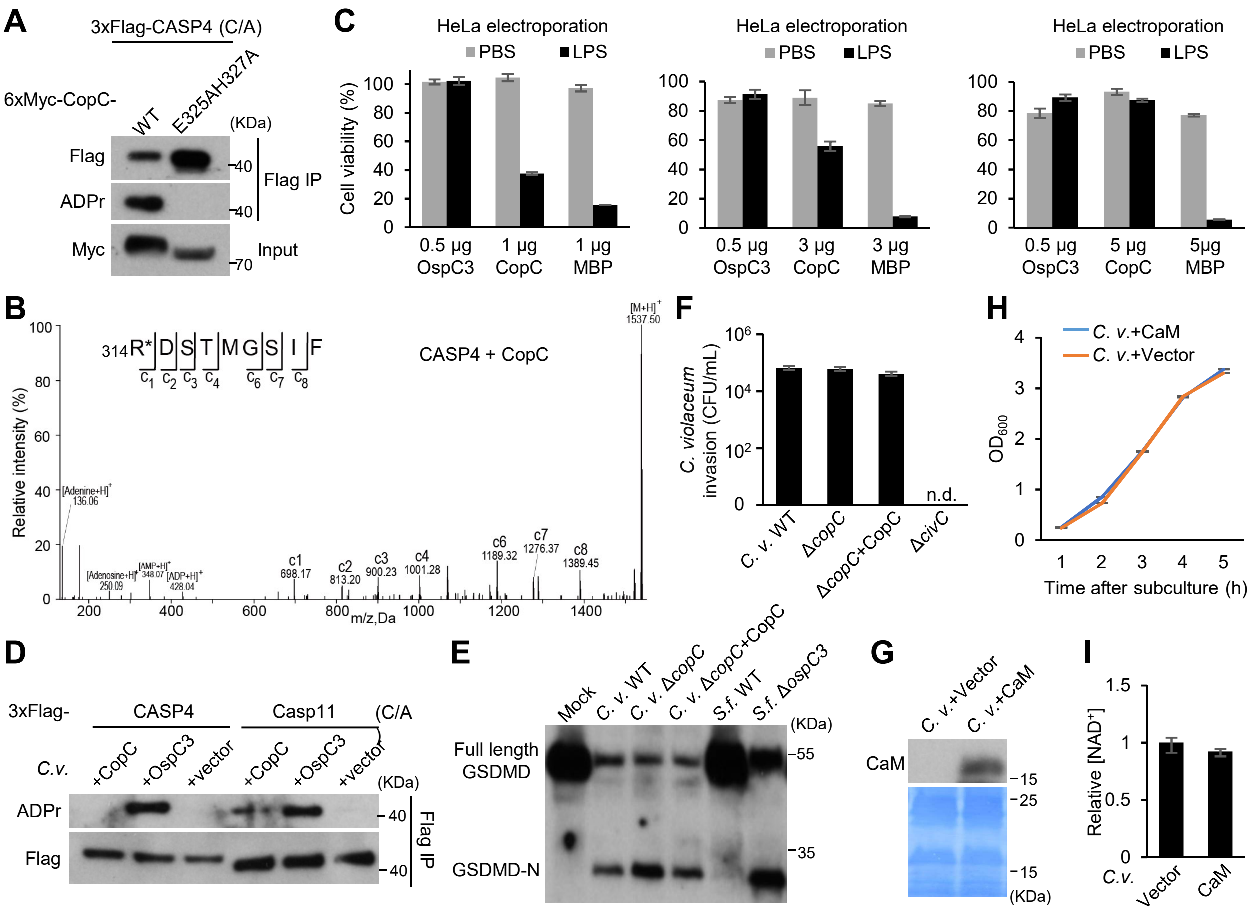

FIG S2 (A) Caspase-4 ADP-riboxanation by overexpressed CopC. 293T cells were co-transfected with 3xFlag-caspase-4 (p30 form, C258A) and 6xMyc-CopC (WT or E325A/H327A) mutant. Cell lysates were subjected to anti-Flag immunoprecipitation (Flag IP) followed by immunoblotting as indicated. Immunoblotting of total lysates (Input) shows the expression of CopC. (B) Mass spectrometry of CopC-modified caspase-4. Caspase-4 was reacted with excessive CopC *in vitro* and then subjected to EThcD mass spectrometry. Shown is the tandem mass spectrum of the modified caspase-4 peptide $_{314}$RDSTMGSIF. (C) HeLa cells were electroporated with LPS together with indicated amounts of purified OspC3, CopC, or MBP proteins. ATP-based cell viability was determined 2 h post-electroporation. Data are presented as means ± SD of three individual replicates. (D) 293T cells transfected with 3xFlag-caspase-4 or -11 (p30 form, C/A) were infected with *C. violaceum* Δ*copC* complemented with CopC, OspC3, or an empty vector. Lysates of infected cells were subjected to anti-Flag immunoprecipitation followed by immunoblotting. (E) HeLa cells stably expressing caspase-4 and GSDMD were infected with *C. violaceum* (WT or a *copC* deletion/complementation strain) or *S. flexneri* (WT or Δ*ospC3*). Cell lysates were immunoblotted with anti-GSDMD antibody. GSDMD-N fragment represents the cleaved active form. (F) HeLa cells were infected with *C. violaceum* WT, Δ*copC*, or CopC complementary strain. Bacterial invasion was determined by counting the numbers of colonies recovered from HeLa cells at 1 h post-infection (means ± SD from three determinations). (G to I) CaM expression in *C. violaceum* has no effect on bacterial NAD^+ content and growth. WT *C. violaceum* was transfected with an empty vector or a plasmid expressing CaM. Bacterial lysates were subjected to anti-CaM immunoblotting and Coomassie Blue-staining (loading control) (G). Overnight bacteria cultures were transferred to fresh medium (1:33), followed by optical density measurements at 600 nm (OD_{600}) at the indicated time points (H). NAD^+ contents in bacterial lysates were assayed using HPLC-MS and relative NAD^+ concentrations were shown as means ± SD from three determinations (I).
