# Supplementary material for: Calmodulin Binding Activates Chromobacterium CopC Effector to ADP-Riboxanate Host Apoptotic Caspases
Source: mBio. 2022 Apr 21;13(3):e00690-22. doi: 10.1128/mbio.00690-22 (PMC9239266; doi:10.1128/mbio.00690-22)
Supplement: FIG S3 [file mbio.00690-22-sf003.pdf]

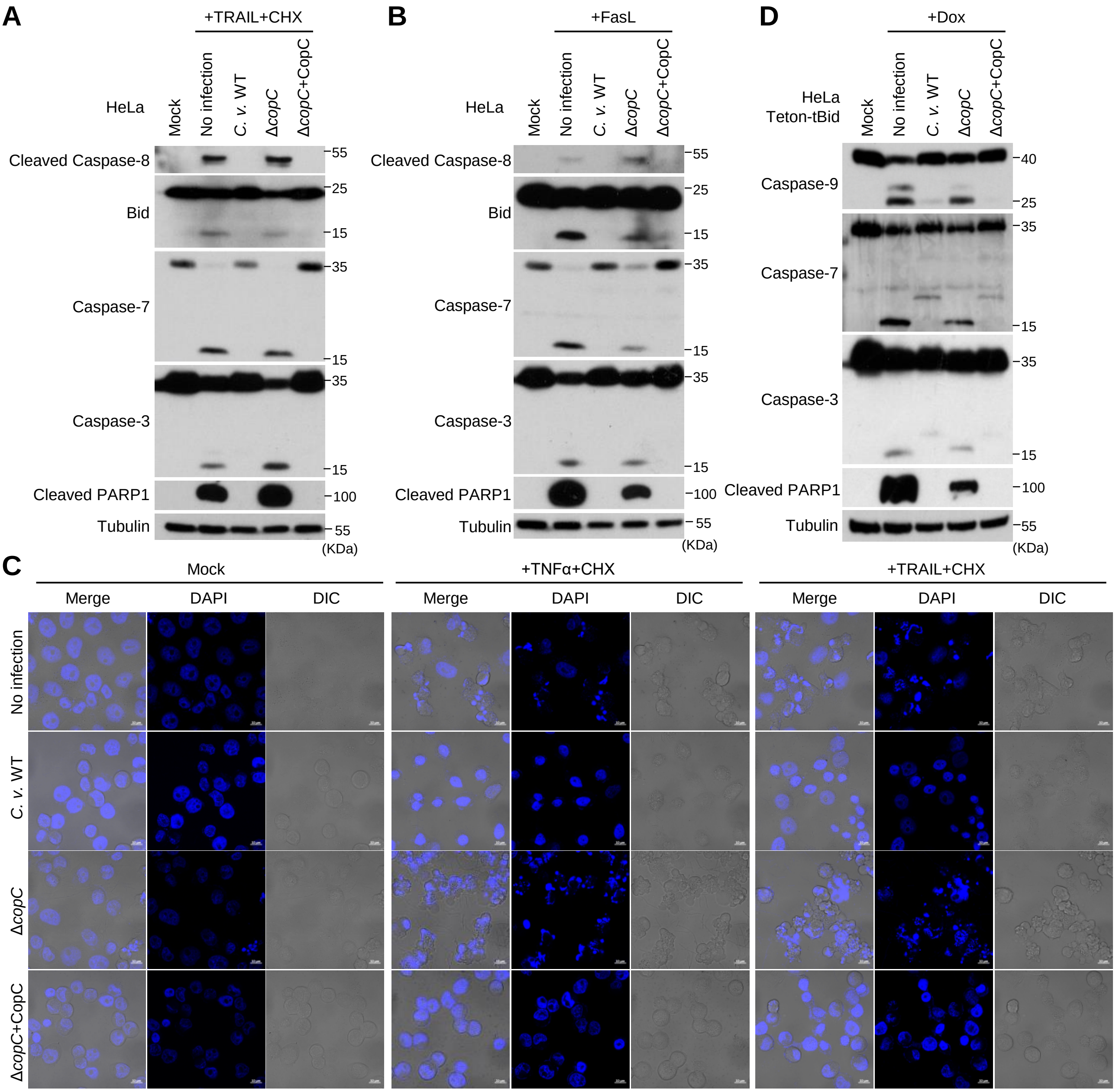

FIG S3 (A to C) Blocking the extrinsic apoptotic pathways by CopC during *C. violaceum* infection. HeLa cells were infected with *C. violaceum* WT, Δ*copC*, or Δ*copC* complemented with CopC; 1 h post-infection, cells were stimulated with 200 ng/mL TRAIL plus 100 μg/mL CHX (A and C), 1 μg/mL FasL (B), or 30 ng/mL TNFα plus 100 μg/mL CHX (C). The immunoblotting in (A and B) was performed similarly as that in Fig. 2B. To examine apoptotic morphology, nuclei of fixed cells were stained with 4′,6-diamidino-2-phenylindole (DAPI). Representative fluorescent and bright field (DIC) cell images are shown (scale bar, 10 μm). (D) Blocking the intrinsic apoptotic pathway by CopC during *C. violaceum* infection. HeLa cells stably expressing truncated Bid (tBid) under a doxycycline (Dox)-inducible promoter were infected as in (A). 1 h post-infection, cells were treated with 1 μg/mL Dox and analyzed by immunoblotting as shown.
