# Supplementary material for: Calmodulin Binding Activates Chromobacterium CopC Effector to ADP-Riboxanate Host Apoptotic Caspases
Source: mBio. 2022 Apr 21;13(3):e00690-22. doi: 10.1128/mbio.00690-22 (PMC9239266; doi:10.1128/mbio.00690-22)
Supplement: FIG S4 [file mbio.00690-22-sf004.pdf]

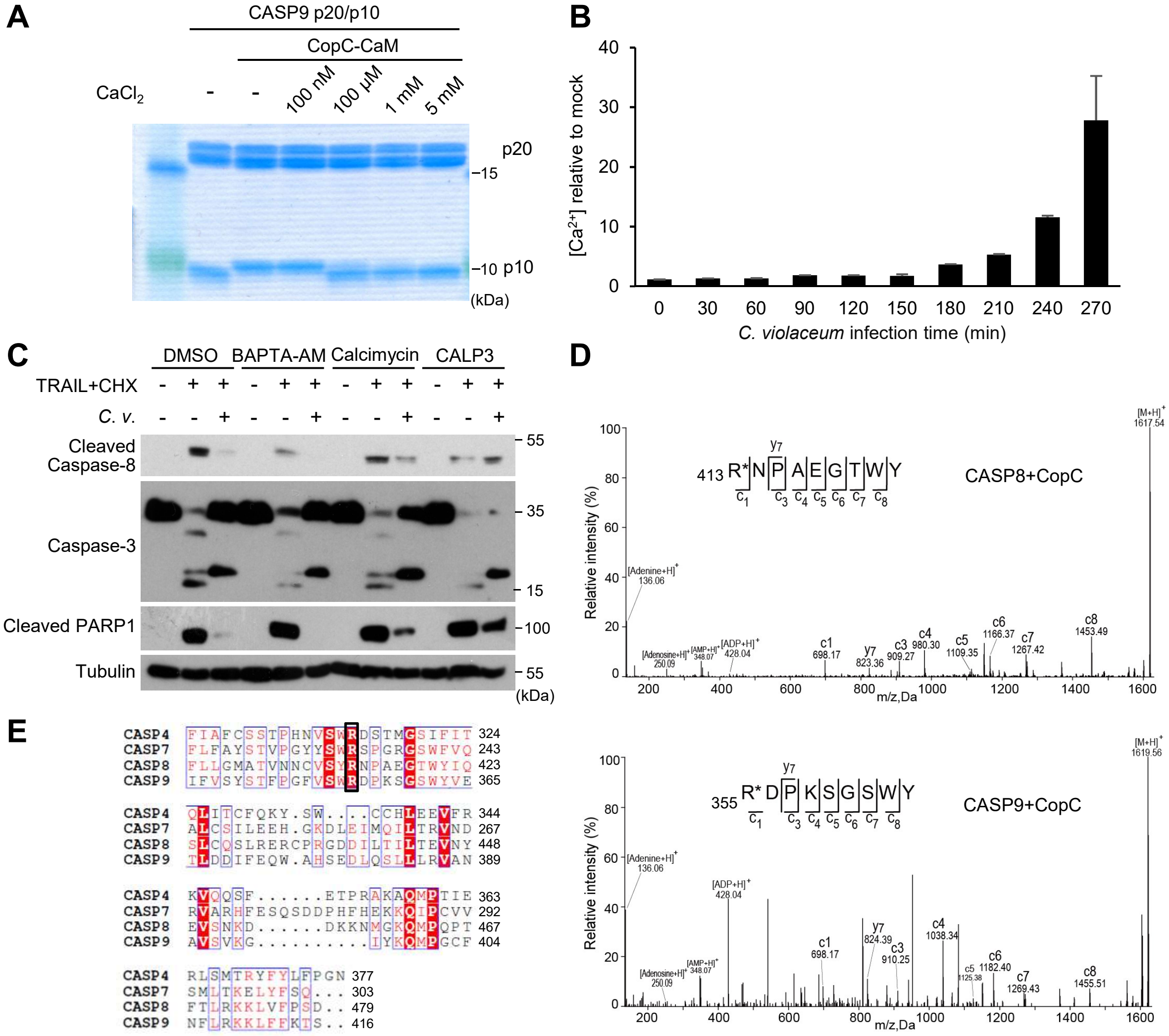

FIG S4 (A) *In vitro* reconstitution of caspase-9 (p20/p10) ADP-riboxanation by the CaM-CopC complex in the presence of different concentrations of $CaCl_2$. The assay was performed similarly as that in Fig. 3A. (B) HeLa cells loaded with Fluo-4 AM were infected with WT *C. violaceum*. The fluorescence intensities were measured at the indicated time points post infection. Calcium concentrations were calculated (see Materials and Methods) and the relative calcium concentrations (compared to uninfected cells) are shown. (C) HeLa cells were loaded with 10 μM BAPTA-AM (pre-mixed with 0.04% pluronic F-127 and added 1 h prior to infection), 2 μM Calcimycin, or 200 μM CALP3 and then infected with WT *C. violaceum*. 1 h post-infection, cells were stimulated with 200 ng/mL TRAIL plus 100 μg/mL CHX for 90 min. The immunoblotting was performed similarly as that in Fig. 2B. (D) EThcD mass spectrometry analysis of CopC-modified caspase-8 (upper) and caspase-9 (lower). Tandem mass spectra of the modified caspase-8 peptide $_{413}$RNPAEGTWY of CASP8 and caspase-9 peptide $_{355}$RDPKSGSWY are shown. (E) Multiple sequence alignment of the p10 subunits of different caspases. ClustalW2 and ESPript 3.0 were used to derive and display the alignment, respectively. The conserved arginine modified by CopC is highlighted by a black rectangle.
