# Supplementary material for: Calmodulin Binding Activates Chromobacterium CopC Effector to ADP-Riboxanate Host Apoptotic Caspases
Source: mBio. 2022 Apr 21;13(3):e00690-22. doi: 10.1128/mbio.00690-22 (PMC9239266; doi:10.1128/mbio.00690-22)
Supplement: FIG S5 [file mbio.00690-22-sf005.pdf]

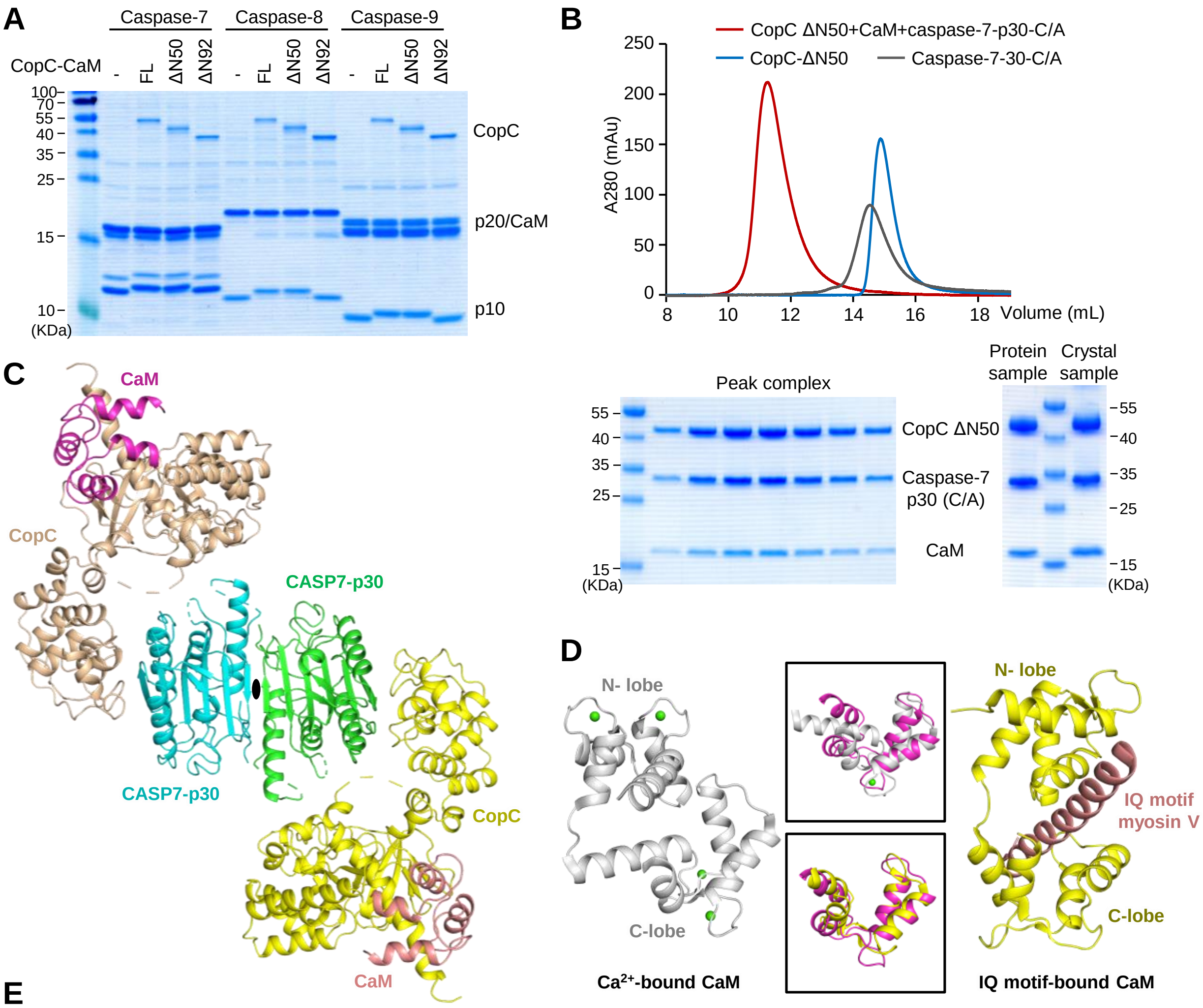

E

	PDB chain	Z score	RMSD (Å)	Aligned residues	Identity (%)	Molecule name
1	6v85-F	3.9	2.6	45	13	RNA-directed RNA polymerase L
2	5daz-A	3.5	3.4	96	10	DNA-acting ADP-ribosyltransferase Scabin
3	2jr5-A	3.5	3.1	60	12	UPF0350 protein VC_2471
4	1wfx-A	3.5	5.7	91	10	RNA 2'-phosphotransferase
5	6l7r-A	3.1	2.5	48	17	Putative spindle pole body component alp6 protein
6	2vse-A	3.1	3.6	110	9	Mosquitocidal toxin
7	4tlv-A	3.0	3.6	96	8	ADP-ribosylating toxin CARDS
8	2jqn-A	3.0	3.0	83	10	Hypothetical protein from Caulobacter crescentus
9	5cwc-A	2.9	4.9	63	6	Designed helical repeat protein
10	2dbh-A	2.7	3.4	55	2	C-terminal CARD-like domain of death receptor-6

F

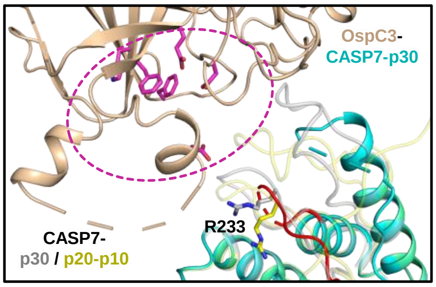

FIG S5 (A) Functional analyses of CopC N-terminal truncation mutants. The p20/p10 form of caspase-7, -8 or -9 were reacted with CopC (full-length (FL), ΔN50, or ΔN92) at the molar ration of 10:1 in the presence of CaM. (B) Gel-filtration chromatography analyses of CopC ΔN50, CaM and caspase-7 p30 complex formation. The peak eluted from the column, the protein samples used for crystallization as well as the crystal samples were assessed by Coomassie-stained SDS-PAGE gels. (C) A cartoon scheme of the 2:2:2 CaM-CopC-CASP7 complex structure. The dimeric ternary complex is mediated by caspase-7 molecules from two neighboring asymmetric units. The 2-fold crystallographic axis is shown. (D) Cartoon models of Ca^{2+}-bound CaM alone (PDB code: 1CDL, left) and Ca^{2+}-free CaM in complex with the IQ motif of myosin V (PDB code: 2IX7, right). Ca^{2+} are shown as green sphere. The middle shows CaM-C lobe in the ternary complex (magenta) overlaid with that in the Ca^{2+}-bound CaM (upper) or Ca^{2+}-free CaM (lower). (E) Dali search results of CopC-NTD structure. (F) Close-up view of the deduced NAD^+-binding site in CopC-NTD and its position relative to the R233-containing loop of CASP7. Structures of isolated CASP7-p30 dimer (PDB code: 1K88) and CASP7-p20/p10 dimer (PDB code: 1K86) were superposed onto that of CASP7 in the ternary complex. The deduced NAD^+-binding site in CopC-NTD is highlighted by a magenta dash-line box with binding residues in stick models.
